# Well-Being of the Baltic Herring and Bycatch Fish Species from FAO Major Fishing Areas 27 According to Microplastic Pollution

**DOI:** 10.3390/ani15162381

**Published:** 2025-08-13

**Authors:** Paulina Piskuła, Aleksander Maria Astel

**Affiliations:** Department of Environmental Chemistry and Toxicology, Institute of Geography, Pomeranian University in Słupsk, 22a Arciszewskiego Str., 76-200 Słupsk, Poland

**Keywords:** pollution with microplastics, the Baltic Sea, principal component analysis, biometric features, Baltic cod, flounder, sprat, long-spined bullhead, lumpfish

## Abstract

Condition factors are essential in fishery science. They provide insights into fish well-being according to the impact of environmental stressors. The Fulton condition factor (K) and hepatosomatic index (HSI), as well as two new indices (GILSI and GITI), were used to assess the well-being of herring and bycatch species (cod, flounder, sprat, long-spined bullhead, lumpfish) from the Baltic Sea. For *Cyclopterus lumpus* and *Taurulus bubalis*, K and HSI values were reported for the first time. Fish from different basins differed according to biometric measures (weight: 6.09–750.12 g; length: 8.6–41.8 mm) and condition indices (mean K: 1.19; HSI: 0.02; GITI: 0.04; GILSI: 0.03). Microplastics (MPs) were found in all species (21% (sprat)–79% (lumpfish)), averaging 3.1 items/fish. The organ-wise analysis revealed that 46% of the gills, 16% of the livers, and 38% of the gastrointestinal tracts were contaminated with MPs. The relationship between these indices and MPs abundance in the organs of herring was examined using PCA, revealing a negative impact of MPs on fish health. The morphometric features and the chemical composition of MPs were also detected. The dominant size ranges were 0.11–0.5 mm (30%) and 1.01–5 mm (28%). Blue fibers were the most common.

## Simple Summary

The abundance of microplastics was determined in the gills, the gastrointestinal tract, and the liver of six marine fish species (Baltic herring, sprat, cod, flounder, long-spined bullhead, and lumpfish) collected from the southern and central Baltic Sea. Microplastics were analyzed in terms of their quantity, size, shape, color, and chemical composition. Four health indices were applied to assess fish well-being according to microplastic pollution. For *Cyclopterus lumpus* and *Taurulus bubalis*, K and HSI values were reported for the first time. Principal component analysis revealed a negative impact of microplastics on the health condition of the Baltic herring.

## 1. Introduction

Fish play a crucial role in the aquatic ecosystems, influencing their resilience and dynamics of trophic structures [1]. Fish contribute to the circulation of carbon (C), nitrogen (N), and phosphorus (P) through processes such as consumption, assimilation, and digestion [2]. Fish constitute a significant food source for large predators [3] and stimulate primary production [4] by releasing nutrients into the environment in inorganic (e.g., excretion) and organic (e.g., egestion) forms.

Despite their wide distribution, fish populations are geographically diverse due to their fragility in the face of environmental stressors. Several fish species can act as bioindicators, since their diversity reflects various impacts on aquatic systems [5,6]. Despite their ecological importance, fish also play a key role in recreation, global and local economies, and human nutrition, being a rich source of proteins, vitamins, minerals, and omega-3 fatty acids [7,8,9].

Marine reservoirs belong to the most polluted ecosystems in the world [10]. Industrial and domestic wastewaters (rich in pathogenic microorganisms, nutrients, pharmaceuticals, metals, and metalloids, etc.), as well as plastic debris, are directly released to aquatic ecosystems [11], exposing organisms to a variety of anthropogenic stressors [12]. Pollution and global climate change (increase in water temperatures, changes in salinity, and ocean acidification) disrupt aquatic ecosystems by affecting fish metabolism, reproduction, and distribution, and triggering health issues such as hormonal imbalances and tissue damage [13,14,15].

The release of wastes rich in MPs dominates among the pressures exerted by humans on water ecosystems. Smaller MPs (primary type) enter aquatic environments through sewage systems [16]. Larger plastic items (secondary type) degrade into MPs, typically with a diameter smaller than 5 mm [17]. MPs carry or leach a variety of xenobiotics, such as flame retardants [18], plasticizers [19], antioxidants [20], UV stabilizers [21], and pigments [22] used in polymer production, as well as persistent organic pollutants, metals, pharmaceuticals, and biotic pathogens [23,24,25,26].

MPs have been identified at all trophic levels of fish fauna [27]. Due to their varied colors, sizes, and shapes, plastic debris resembles natural food. As a result, fish may ingest it (intentionally or accidentally) [28,29]. The occurrence of MPs in the muscles, the liver, the gills, the digestive glands, and the circulatory system of fish has been confirmed [30,31,32,33]. Once ingested into the gastrointestinal tract, MPs remain in it or can be ultimately expelled; however, some items can also be translocated to different tissues and organs [30]. The ingestion of plastic items facilitates the blocking or injuring of the gastrointestinal tract [34], as well as the malfunctioning of other tissues or organs due to the leaching of toxins. Xenobiotics, as well as MPs, also enter the fish body through the gills and then migrate via the bloodstream [35]. The gills are highly sensitive to physical and chemical changes in the aquatic environment. This makes them a valuable indicator of waterborne toxic substances [36]. Fortunately, fish are capable of developing numerous defense mechanisms that can prevent the negative effects of toxins on the gills. These include mechanisms involving morphological changes in the gills, such as interlamellar edema [37], epithelial proliferation, and desquamation of the pavement epithelium [38].

The aforementioned environmental stressors often act synergistically, which exacerbates their negative effects on marine fish. In this context, physiological indicators are commonly used as early warning signs of declining well-being [39,40], potentially preceding impairments in reproduction or survival [41,42]. The most commonly used physiological indicators are Fulton’s condition factor (K factor) and the hepatosomatic index (HSI) [43]. K factor expresses the ratio of the fish’s mass to its length [44]. It is used to measure the reserve of fish energy and indicates the general health status of fish [45]. A high value of K suggests that the fish is well-nourished and in good physical condition, while a low value usually indicates feeding problems, environmental stress, or health issues [46]. The HSI expresses the ratio of the liver mass to the total mass of the fish as a percentage [47]. It reflects the accumulation of lipids in the liver [48]. Since it is used to assess the condition of the liver, it indicates the physiological state of fish, their ability to accumulate energy, and the impact of environmental and dietary factors [49].

The determination of biometric-based health indices, as well as the evaluation of the gastrointestinal tract and the gills, can be an important method for a comprehensive assessment of fish health conditions. However, according to our knowledge, in previous studies on fish health assessment, there was a lack of comparative evaluations of the proportions of the gill mass and gastrointestinal tract mass to the total body mass of fish, and hence, the current research study contributes to a better understanding of fish population health. Although the presence of MPs in various tissues and organs of fish has been confirmed, there are still research gaps concerning the impact of MPs on physiological indicators of wild marine species.

Since MPs items pose a threat to fish health, the aims of this study were (i) to analyze the biometric features of fish from the Baltic Sea; (ii) to quantify the abundance of MPs in the gastrointestinal tract, the gills, and the liver; (iii) to assess the well-being of all species by evaluation and comparison of fish condition parameters based on the K and HSI indices, as well as on the ratios of gill mass (GILSI) and gastrointestinal tract mass (GITI) to total body mass; (iv) to explore the relationships between biometric features and condition parameters for Baltic herring according to fishing season and fishing zones by the use of multivariate analysis, and, finally, (v) to characterize MPs according to size, color, shape, and chemical type.

## 2. Materials and Methods

### 2.1. Study Area

The Baltic Sea (BS) is the largest brackish sea, characterized by unique hydrographic, geological, and biological properties [50]. The sea catchment area is located in Northern Europe and is surrounded by nine countries: Poland, Germany, Denmark, Sweden, Finland, Estonia, Latvia, Lithuania, and Russia [51]. The sea stretches from 53° N to 66° N latitude and from 10° E to 30° E longitude [52]. The surface area is 377,000 km^2^, while the average depth is 55 m [53]. The BS is characterized by low to medium salinity levels, modified by excessive river runoff and limited water exchange with the North Sea through the Kattegat and the Danish straits [54]. The sea is stratified, with a vertical structure consisting of two water masses: brackish surface waters with a salinity of approximately 7–8 psu and deep waters with a salinity between 11 and 13 psu in the Baltic Proper [55]. Low salinity, increased water temperature, and nutrient discharge via river runoff cause an increase in the intensity of algal blooms, often responsible for oxygen poverty and threatening marine life [56]. The diversity of coastal zones includes archipelagos, fjords, cliffs, continents, bays, lagoons, compensatory coasts, deltas, and long, narrow bays, creating important habitats for marine biota and recreational areas for humans [57].

The sea is isolated [58], semi-enclosed [59], shallow [59], and needs 25–40 years to replace its whole water [50]. The enclosed characteristic of the BS results in a unique ecosystem that makes it especially fragile to human activities [60]. The BS is regarded as one of the most polluted seas in the world [61]. More than 80% of pollution is released through land-based activities, while less than 20% is due to shipping, fishing, exploitation of marine resources, and other activities [50]. Industrial, agricultural, and municipal wastes are directly or indirectly discharged into the sea. The majority of hazardous substances enter the waters of the southern and eastern BS through large rivers, including the Oder, Vistula, Niemen, and Neva. The total annual load of nutrients released into the BS basin from food, fodder, and fertilizers averages approximately 2100 and 340 kilotons of nitrogen and phosphorus, respectively [62]. It results in eutrophication, leading to a reduction in oxygen at the sea bottom [63].

### 2.2. Baltic Sea Pollution with MPs

MPs pose a significant threat to the BS ecosystem. Among the many potential sources of MPs released to the sea, the most important are municipal wastewater [64], rivers [65], port activities [66], tourism [67], and fishing and shipping [68]. Consecutively, MPs have been detected in the surface waters of the BS [69], in bottom sediments [70], as well as in beach sediments [71]. The presence of MPs in various parts of the BS ecosystem indicates their widespread distribution across the region, which poses a significant threat to the health of marine organisms. MPs are accidentally or directly ingested by marine organisms, such as fish from the southern BS [72,73], the Finnish waters [74], and the Bornholm Basin [75], which leads to disruptions in their functioning, death, and biomagnification along the food chain.

### 2.3. Fish Communities in the Baltic Sea

The fish community of the BS with the Kattegat includes about 200 species [76]. Fish fauna contains both freshwater and marine species [77]. The Baltic fish biomass is dominated by cod (*Gadus morhua*), herring (*Clupea harengus*), and sprat (*Sprattus sprattus*). In the last 30–40 years, the wealth of dominant fish species has undergone many changes [78]. *Gadus morhua* biomass remained high in the late 1970s and early 1980s, before declining over the next 15–20 years. The decrease in cod population was associated with overfishing, low reproductive success, climatic and hydrographic changes, and a high number of predators of cod eggs and larvae [79]. The biomass of sprat and herring also fluctuates, mainly due to changes in the abundance of their predators, as well as climatic conditions and interspecies competition for food [80]. Numerous species migrate into the BS from the North Sea, including whiting (*Merlangus merlangus*), European anchovy (*Engraulis encrasicolus*), mackerel (*Scomber scombrus*), and gray mullet (*Liza ramada*). However, due to unfavorable environmental conditions, migratory species are unable to establish self-sustaining populations within the Baltic. The BS also hosts migratory fish species of significant commercial value, particularly salmon (*Salmo salar*), trout (*Salmo trutta*), and eel (*Anguilla anguilla*). Coastal regions of the BS are predominantly inhabited by freshwater fish species, including perch (*Perca fluviatilis*), roach (*Rutilus rutilus*), bream (*Abramis brama*), and pike (*Esox lucius*). Freshwater species are more prevalent in areas of lower salinity, such as the northeastern BS, including large bays and lagoons [81].

### 2.4. Detailed Description of the Studied Species and the Fish Samples’ Collection Protocol

The study included 6 marine fish species (the Baltic herring, the Baltic cod, the flounder, the long-spined bullhead, the lumpfish, and the sprat).

Baltic herring (*Clupea harengus*) is one of the most dominant fish species in fish processing all over the world [82]. Herring plays a key ecological role, acting as a connection between zooplankton and predatory fish [83]. Herring is a pelagic fish. It lives in the open waters of oceans and seas, typically near the water’s surface. Individuals live in shoals, which may provide them with protection from predators [84]. The main food for herrings is plankton, such as small crustaceans, fish larvae, and other tiny planktonic organisms [85]. Herrings are sensitive to environmental changes such as chemical pollution, water temperature, and food availability. Their presence, abundance, and health condition can provide information concerning the state of the marine ecosystem, making them useful indicators for monitoring the marine ecosystem status [86].

Cod (*Gadus morhua*), sprat (*Sprattus sprattus*), and flounder (*Platichthys flesus*) are three popular commercial fish species naturally present in the BS. Cod live at depths ranging from 20 to 200 m [87] and occur both in open waters and along shallow coastlines [88]. The diet of cod primarily consists of fish such as herring and flounder, as well as crustaceans, mainly shrimp and mussels [89]. Sprat is the most important fish in the food webs of the open part of the BS and constitutes the largest part of the fish catches. The species is distributed throughout most of the BS; however, the brackish environmental conditions allow for its reproduction mainly in the open Baltic, the western and central Gulf of Finland, and in some adjacent areas. The diet of sprat is mainly based on plankton, such as small crustaceans and fish larvae [90]. The flounder has an important commercial potential. It feeds in shallow, coastal areas during summer and moves out to deeper areas in winter [91]. Juveniles feed on plankton and insect larvae, while adults feed on mollusks, crustaceans, and small fish [92].

The study also includes two non-commercial fish species, the long-spined bullhead (*Taurulus Bubalis*) and the lumpfish (*Cyclopterus lumpus*), which were caught as bycatch. Although these species do not have significant commercial value, they play a crucial role in the marine ecosystem. The long-spined bullhead is a small, bottom-dwelling fish that feeds mainly in coastal regions with rocky bottoms [93]. It feeds on small invertebrates, helping to control their populations [94]. Lumpfish belong to a benthic fish species that feed on crustaceans, small fish, and jellyfish. The meat of the females is unpalatable, while lumpfish roe is sold as imitation caviar [95].

**Figure 1:**
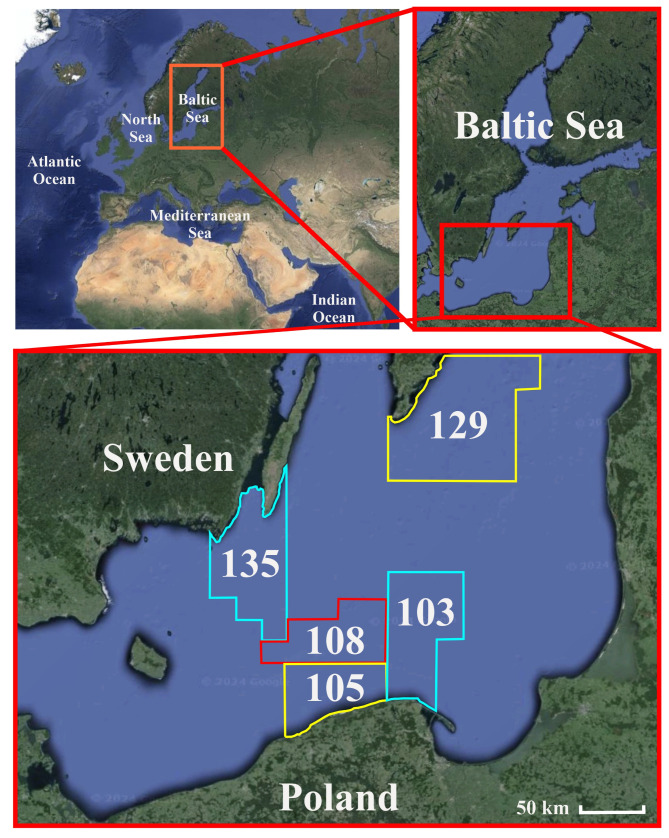
The sampling sites of fish collection in the southern BS, including fishing zones 103 (Władysławowskie), 105 (Ustecko-Łebskie), 108 (Słupsk Gutter), 129 (Gotlandic), and 135 (Kalmarskie), located within FAO fishing area 27.III.d.25 (the colors of frames surrounding fishing zones used have no meaning).

Fish were acquired seasonally (November 2021; February, September, and October 2022; April, October 2023; January 2024) according to permitted fishing periods by a commercial fishing vessel within area 27 of the FAO Major Fishing Areas for Statistical Purposes, sub-area III.d.25 (fishing grounds: 103, 105, 108, 129, 135), presented in Figure 1. Commercial fishing was adjusted to the permitted fishing period to protect spawning and juvenile fish, and hence, healthy and fully developed specimens were expected. Neither water sampling nor assessment of the food supply efficiency, nor any preparatory activities, were carried out by fishermen on board. Once the fish acquired during the commercial fishing event were delivered to the company, a sample of Baltic herring and bycatch specimens was donated to research and transported to the laboratory. Every batch was frozen at −20 °C until further analysis for the presence of MPs and morphometric measurements according to a previously published procedure [96]. A total of 257 fish acquired in the period between November 2021 and January 2024 were analyzed in the study.

### 2.5. Sample Preparation

Once the fish had thawed at room temperature, the total body length (centimeters, TL) and total body mass (grams, TW) of each individual were determined. Moreover, a rough visual assessment of the specimens was performed, and neither anatomical nor skin diseases were detected. All specimens were washed in deionized water (HLP 10 UV, Hydrolab, Straszyn, Poland). The organs (the gastrointestinal tract, the liver, and the gills) were dissected and weighed separately using a digital analytical scale with an accuracy of ± 0.0001 g (Ohaus PX225D, Parsippany, NJ, USA). The prepared organs were placed in dry glass beakers. Samples of fish organs were digested using 10% potassium hydroxide to isolate MPs. Once the digestion process was completed, samples were directly filtered onto 0.11 μm pore size Whatman No. 1001-090 Filter paper (GF/F Whatman™, Pittsburgh, PA, USA).

All suspected plastic items were assessed following the protocols recommended by Hidalgo-Ruz et al. [97], Crawford and Quinn [98], and Zobkov and Esiukova [99]. Items with no visible tissue or cell structure, of relatively uniform color distribution along the particle, and fibers with homogenous diameters along their length were counted as MPs. The other objects were recognized as minerals and excluded from further stages of the analysis. An analogical procedure for the microscopic determination of MPs was applied by Wang et al. [100]. Consequently, basic morphometric features (the longest dimension, color, and shape) of all items qualified as plastic were determined using MotiConnect 1.5.9.10—build-171,215 software. The identified MPs were divided into three morphological types: fragments, fibers, and pellets. MPs were also categorized into five groups according to size: <0.1, 0.11–0.5, 0.51–1, 1.01–5, and >5 mm.

Plastic particles larger than 0.5 mm were qualitatively analyzed by an ATR FT-IR spectrometer (Thermo Scientific, Nicolet iS5 with ATR diamond crystal, Waltham, MA, USA). Due to the technical limitation of the ATR FT-IR device, smaller items cannot be qualitatively analyzed since the signal obtained during the analysis of samples with narrow cross-sections is usually insufficiently intense, and hence, the acquisition of the characteristic absorption bands is challenging. Moreover, although some fibers exceed 0.5 mm in length, their width is often too small to be effectively analyzed using ATR FT-IR, which prevents the acquisition of reliable absorption spectra. As a result, approximately 15% of the total items classified as MPs were subjected to polymer identification by FT-IR spectroscopy. Despite this disadvantage, it was assumed that the percentage contribution of polymer types is also valid for MPs of smaller sizes, since larger items usually disintegrate into smaller size fractions. Moreover, the quantitative assessment of every item was not a major aim of the study. According to EU recommendations, spectra that matched over 70% of the standard database (Hummel Polymer Sample Library) were directly acceptable as MPs composed of particular polymer types [101]. A detailed description of the extraction procedure, as well as methodological aspects of the ATR FT-IR measurements, are described in already published references [102].

### 2.6. Contamination Prevention

Clean cotton laboratory aprons and nitrile gloves were worn during all stages of the procedure. The possibility of airborne or external sample contamination with MPs was minimized by laboratory work by using glass, aluminum, and stainless-steel materials. Samples were kept covered at all times. The above contamination prevention method was described in detail in a previous study [96]. To explore the possibility of external or airborne contamination throughout the process, a procedural blank sample was prepared for each fish sample analyzed. In total, 257 procedural blank controls were conducted. Each blank consisted of an empty glass beaker processed using the same protocol as the actual samples, but without any biological material. The blanks were treated with 10% KOH and placed in an oven under the same conditions as the fish samples, followed by filtration using Whatman filters. The resulting filters were then transferred to Petri dishes for further analysis. This approach allowed for the detection and quantification of any background contamination introduced during sample preparation. No MPs contamination was detected in the blank samples, although, due to the binary and discrete characteristics of MPs determination, LOD and LOQ were set to one.

### 2.7. Fish Health Condition

Fish condition status was assessed by Fulton’s condition factor (K) (Equation (1)), hepatosomatic index (HSI) (Equation (2)), the ratio of gill mass to the total body mass of fish (GILSI) (Equation (3)), and the ratio of gastrointestinal tract mass to the total body mass of fish (GITI) (Equation (4)), using formulas summarized below:

K = (total fish weight [g] × 100)/(total fish length [cm]^3^) (1)

HSI = liver weight [g]/total fish weight [g](2)

GILSI = gills weight [g]/total fish weight [g](3)

GITI = gastrointestinal tract weight [g]/total fish weight [g](4)

A literature review indicated that researchers commonly apply the K factor using various assessment classifications [103]. Although studies provide results for this factor, cross-species comparisons remain difficult. Therefore, an additional novelty of the current study involved standardizing the K factor to unity, which, in our opinion, enables approximate comparison of the K factor across different species. Moreover, two novel indices, GILSI and GITI, were proposed to enhance fish health assessment methods. For five of the six fish species, a complete set of indices was calculated, enabling a more comprehensive evaluation of their health condition. However, for sprat, due to the small size of the fish and the associated difficulty in organ isolation, only morphometric measurements of body length and weight were conducted, and thus, only the K condition factor was calculated. For *Cyclopterus lumpus* and *Taurulus bubalis*, K and HSI values are presented for the first time.

### 2.8. Data Analysis

MPs abundance in this study was expressed using two conventions: to compare the abundance of MPs in contaminated fish, the average number of MPs per fish was computed, while the abundance of MPs per organ was expressed as the average number of MPs per type of contaminated organ. The overall average (across all fish species) of MP item occurrence was calculated only for fish with plastic items detected. This approach enables a more detailed assessment of organ-specific accumulation patterns and follows the methodology applied in previous studies on BS fish [73,75,104,105,106].

Each variable was checked for normal distribution using the Shapiro–Wilk test. Since a non-normal distribution was confirmed, the Kruskal–Wallis ANOVA and the median test were used to assess differences in MPs concentrations between species and organs. All tests were analyzed and considered significant at a value of *p* < 0.05. A Spearman correlation coefficient was computed to confirm whether there was any relationship between major biometric characteristics according to species.

Multidimensional data exploration was accomplished by the use of principal component analysis (PCA). Before its use, Bartlett’s sphericity test was computed to check whether the use of PCA would be more beneficial than standard analysis. Once successful verification was accomplished, the PCA model was created, and principal components with an eigenvalue higher than 1 (Kaiser’s criterion) were further analyzed. Both factor loadings and scores were used for visualization purposes. MPs’ abundance, K, HSI, GILSI, and GITI were used to compute the PCA solution, while mass and length descriptors were used as accompanying variables. Statistical analysis was performed with TIBCO Statistica 13.3 (TIBCO, Palo Alto, CA, USA).

The Sankey plot [107] was prepared using an appropriate statistical and graphical package, such as ggplot2 [108] in R [version 4.3.3; R Core Team, 2024], to present the color of plastic items according to the dimension classes.

To allow for the interspecies comparison of the K factor values, their normalization across all species and specimens was executed according to Equation (5):

K_normalized_ = K_computed_ − K_min_/K_max_ − K_min_ (5)

This procedure scales K values to a universal range between 0 and 1, eliminating differences due to morphological variation and providing a single, comparable unit for all species analyzed. The normalized scale makes comparison of fish health conditions, regardless of species and size, possible.

## 3. Results

### 3.1. Fish Biometry

A total of 128 Baltic herring, 46 flounder, 30 Baltic cod, 19 lumpfish, 6 long-spined bullhead, and 28 sprat specimens were analyzed. The range of the total length and basic length of all individuals was 8.6–41.8 cm and 7.1–35.6 cm, respectively, while the range of mass was 6.09–750.12 g. The average total length of the fish according to species was 18.93 ± 2.48 cm, 25.52 ± 4.14 cm, 29.98 ± 4.22 cm, 15.49 ± 1.25 cm, 23.33 ± 3.06 cm, and 11.26 ± 0.92 cm, while the average total body mass was 50.08 ± 15.71 g, 217.13 ± 126.02 g, 284.64 ± 170.69 g, 165.54 ± 39.04 g, 284.61 ± 208.58 g, and 10.03 ± 1.95 g for Baltic herring, flounder, Baltic cod, lumpfish, long-spined bullhead, and sprat, respectively. The range of the mass of the liver, the gastrointestinal tract, and the gills for all individuals was 0.10–21.32 g, 0.04–120.42 g, and 0.51–33.04 g, respectively. Detailed basic statistics, including means, medians, ranges, and standard deviations of morphometric features computed for all specimens according to species, are summarized in Table 1. Because, in rare cases, the isolation of the entire organ was not possible (mainly in some sprat, Baltic cod, and lumpfish specimens), the number of measurements (N) of the morphological characteristics and mass of organs can occasionally be different.

**Figure 2:**
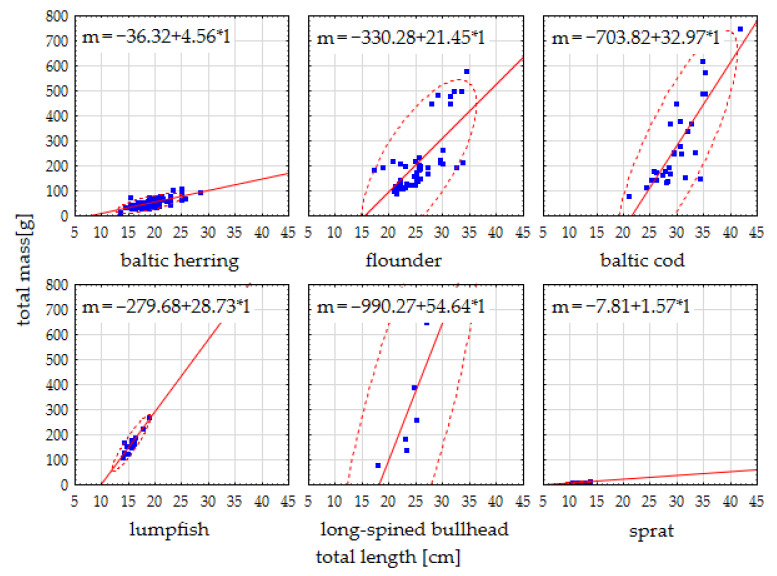
Relation between total length (l) and total mass (m) according to fish species (straight line—regression line; dashed ellipse—a two-dimensional area of normal data distribution at *p* = 0.05).

Except for the long-spined bullhead, a highly significant positive correlation was found between the total length of the body of the fish and their total mass. The values of the significant correlation coefficients were between 0.70 (flounder, *n* = 46; critical value (r_S_crit_) = 0.246) and 0.88 (lumpfish, *n* = 19; r_S_crit_ = 0.391). Detailed values of Spearman correlation coefficients between major biometric characteristics according to species are summarized in Table S1. The score plot of total mass to total length with linear regressions according to species, as well as the range of values demonstrating a two-dimensional area of expected normal data distribution at *p* = 0.05, is presented in Figure 2.

### 3.2. MPs Contamination According to Species and Organs

MPs were found in all examined species. A total of 471 items were found in the gills, the gastrointestinal tract, and the liver of 150 out of 257 individuals (58%). The highest share of specimens with MPs found in at least one out of the three organs was determined in lumpfish (79%), while the lowest was in sprats (21%). The Baltic cod, the flounder, the Baltic herring, and the long-spined bullhead were characterized by a moderate percentage share (57–67%) of MPs.

The highest percentage share of MPs was observed in the gills (45%), a moderate one was observed in the gastrointestinal tract (37%), while the lowest was in the liver (15%), which is in agreement with the expectations. The accumulation of items in the organs differed statistically (H = 68.79, df = 2, *p* < 0.001). Multiple inter-organ comparisons revealed that MP abundance in the liver was statistically lower than that in the gills (*p* < 0.001) and in the gastrointestinal tract (*p* < 0.001). No significant differences in MPs abundance were detected between the gastrointestinal tract and the gills (*p* > 0.05). A detailed statistical assessment of the differences in MPs abundance according to the type of organs across fish species is summarized in Table 2.

The number of MPs ranged from 1 to 12 particles per contaminated fish, with an average of 3.1 items/fish, without taking into account the division into organs and species. Based on the results of the Kruskal–Wallis test computed for all specimens, statistically significant differences in the number of identified MPs according to fish species were found (H = 12.799, df = 5, *p* = 0.02). The lowest number of items was found in contaminated sprat (1.5 items/fish), a moderate number in Baltic cod, Baltic herring, and in long-spined bullhead, (3.0, 2.8, 2.0 items/ fish, respectively), while the highest number was found in flounder (4.3 items/fish) and lumpfish (4.1 items/fish).

### 3.3. Fish Condition Status

**Figure 3:**
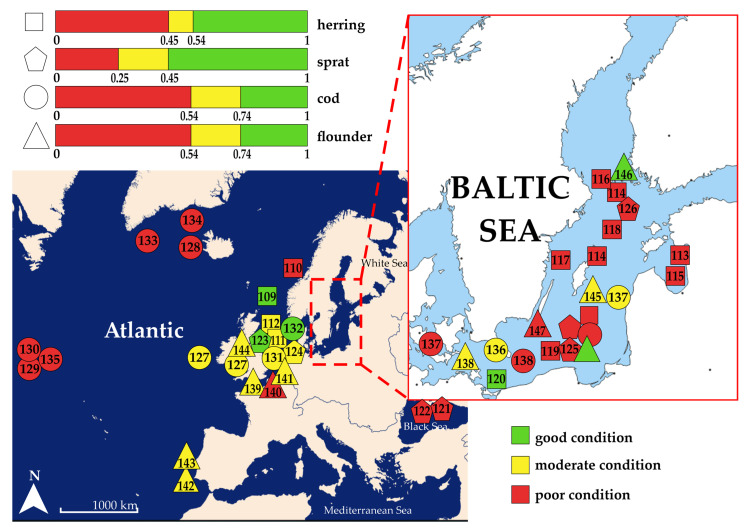
Normalized K factor values according to species based on an intensive literature survey and current data (numbers in shapes correspond to references: good condition; moderate condition; poor condition [109,110,111,112,113,114,115,116,117,118,119,120,121,122,123,124,125,126,127,128,129,130,131,132,133,134,135,136,137,138,139,140,141,142,143,144,145,146,147]; empty shapes correspond to the current study).

The K factor of all individuals was in the range between 0.34 (Baltic cod) and 5.94 (lumpfish). The averaged K factor according to individual species was 0.75 ± 0.22, 1.29 ± 0.59, 0.98 ± 0.31, 4.41 ± 0.49, 1.97 ± 0.86, and 0.69 ± 0.12 for Baltic herring, flounder, Baltic cod, lumpfish, long-spined bullhead, and sprat, respectively. Figure 3 shows the normalized K factor values for the fish species, based on referenced and current data.

The HSI values ranged from <0.01 (Baltic herring) to 0.14 (long-spined bullhead). The lowest average HSI values were observed for herring (0.01), flounder (0.02), and cod (0.03); moderate values were observed for long-spined bullhead (0.06), and the highest for lumpfish (0.09). The GILSI for all individuals was similar, ranging from 0.01 to 0.08 (Baltic herring). The averaged GILSI values for all specimens were rather similar; however, values for individual species were 0.03 ± 0.01 for the Baltic herring and the flounder, 0.03 ± 0.01 for the lumpfish, 0.04 ± 0.01 for the Baltic cod, and 0.04 ± 0.02 for the long-spined bullhead. Similar observations were recorded for the GITI. The mean values of the GITI did not differ significantly according to species. The GITI ranged from <0.01 (herring and cod) to 0.20 (flounder). The lowest average GITI was observed for herring and cod (0.04 ± 0.02); moderate values were observed for flounder (0.05 ± 0.03), while the highest was for lumpfish (0.11 ± 0.02) and long-spined bullhead (0.11 ± 0.06). Detailed basic statistics, including means, medians, ranges, and standard deviations of fish condition indices according to species, are summarized in Table 1 and are presented above.

**Figure 4:**
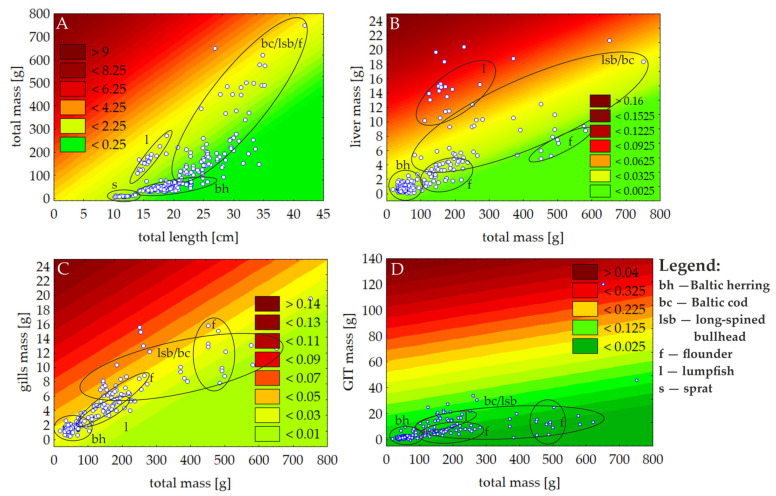
Relation between total length, total mass, and K factor (**A**); total mass, liver mass, and HSI values (HSI) (**B**); total mass, gills mass, and GILS index (GILSI) (**C**); and between total mass, gastrointestinal tract (GIT) mass, and GIT index (GITI) (**D**) according to fish species (bullets—particular specimens, ellipses—surround specimens of the given species).

Based on the proposed fish condition indices, heat maps presenting mutual relations between masses and lengths were created (Figure 4). They enable an assessment of the intraspecimen variation in values according to species. The lowest intraspecies variation in values of the K factor was observed in the Baltic herring and the lumpfish. In both species, all individuals were characterized by rather homogeneous K values across the population. Moderate intraspecimen variation was noted in the sprat, where only a few individuals were characterized by slightly higher K values than the others. The greatest intraspecies variation was observed in the Baltic cod, the long-spined bullhead, and the flounder, as some individuals were characterized by high K values, while others by moderate or low values (Figure 4A). As for the HSI, greater intraspecies variation was observed in comparison to the K factor. The highest variation was noted in the long-spined bullhead and the Baltic cod and a moderate one in the Baltic herring and the flounder, while the lowest was in the lumpfish (Figure 4B). The GILSI exhibited the highest intraspecies variation in comparison to previous indices. The lowest, although not unequivocal, intraspecies variability in the GILSI was observed in the Baltic herring and the lumpfish. All individuals of these two species were within the range of two adjacent areas on the plot, with similar GILSI values across the populations (Figure 4C). Moderate and high variation was observed in the flounder and the Baltic cod and in the long-spined bullhead, respectively, as individuals were distributed across areas corresponding to both low and high GILSI values. In comparison to all other health condition indices, the GITI index exhibited the lowest intraspecies variability for all species. All individuals were located within the same area on the plot (Figure 4D). The smallest GITI variation in individuals was noted for the Baltic herring and the lumpfish, while greater variation was observed for the remaining species.

### 3.4. Multidimensional Analysis

**Figure 5:**
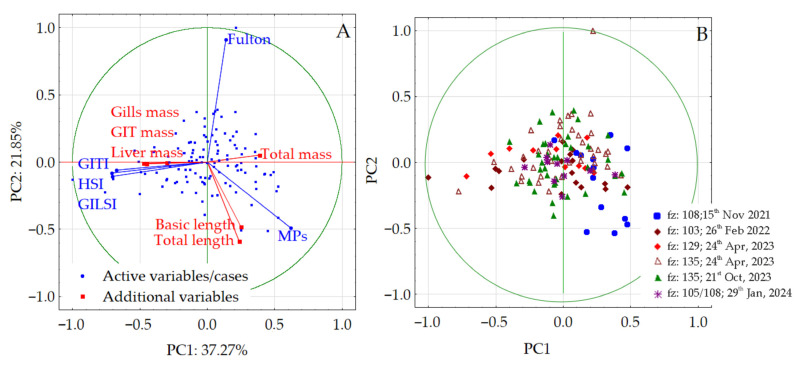
Biplots of PC1 and PC2 loadings and scores with active variables and cases and additional variables (**A**) and principal component scores according to fishing zone (fz) and fishing date (**B**), respectively, computed for the Baltic herring data set.

The relationship between MPs abundance and fish well-being indices was evaluated by the use of PCA. According to the PCA, two principal components with eigenvalues higher than 1 (PC1—1.87, PC2—1.08) were obtained, accounting for 59.12% of the total variance. The first principal component, explaining 37.27% of the total variance, was contributed by HSI, GILSI, GITI, and MPs, while the second, explaining 21.85% of the total variance, was contributed by the K factor and MPs. Within PC1 HSI, GILSI, and GITI exhibited a mutual directly proportional correlation (factor loadings in the range of −0.67– −0.74 of the same sign); however, they were indirectly correlated with MPs (0.56), which also significantly contributed to PC1. PC1 could be accepted as a component presenting the negative impact of MPs on the development of major organs, since decreasing index values corresponded to an increase in MPs abundance. Similarly, as in PC1, the K factor and MPs contribution in PC2 were characterized by opposite signs, suggesting a negative impact of MPs abundance on the well-being of fish, as expressed by the K factor. A biplot of PC1 and PC2 loadings with the corresponding factor scores is presented in Figure 5A, while the separated plot of sample scores according to the fishing zone and fishing date is presented in Figure 5B.

### 3.5. Physical and Chemical Characterization of MPs

The length of MPs items detected in fish organs ranged from 0.006 to 6.94 mm, with an average of 0.84 ± 0.95 mm. Only 4 of the 471 (0.8%) plastic items were larger than 5 mm. Although particles of this length do not necessarily fall within the definition of MPs, due to their negligible contribution, they were included in the analysis of the results. The dominant size range was 0.11–0.5 mm (30%) and 1.01–5 mm (28%), followed by 0.51–1 mm (23%), ≤0.1 mm (18%), and ≥5 mm (1%). In the gastrointestinal tract, the gills, and the liver, the dominant size range was 1.01–5 mm (33%), 0.11–0.5 (33%), and < 0.1 (82%), respectively.

**Figure 6:**
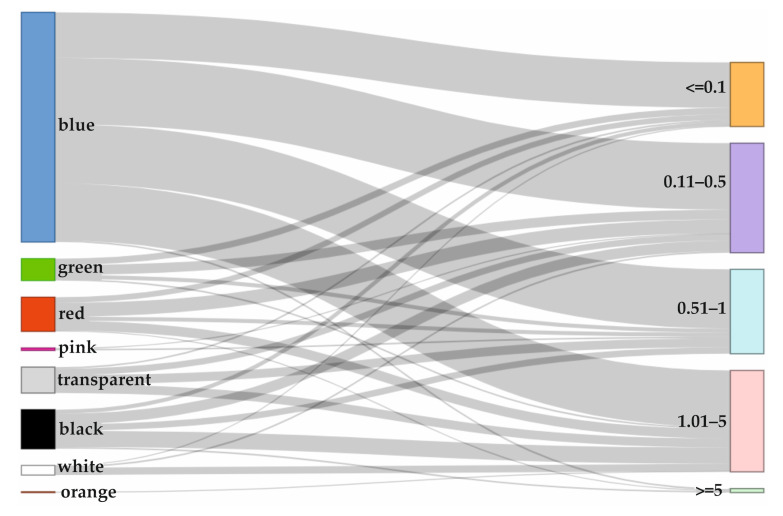
Quantitative distribution of MPs items colors across different size classes, visualized as a Sankey diagram (the colors of size classes have no meaning).

The most dominant color observed was blue (62%), followed by black (12%), red (9%), transparent (7%), green (6%), white (3%), and pink (1%). The quantitative distribution of MPs items’ colors across the different size classes is presented in the form of a Sankey diagram (Figure 6).

Three different types of MPs (fibers, fragments, and pellets) were found in the studied fish species. Fibers were the most prevalent form (70%), followed by fragments (29%) and pellets (1%).

A total of 69 items of size > 0.5 mm out of the 471 MP items (15%) were analyzed using FT-IR ATR to determine their chemical composition. Cellophane accounted for the largest proportion of MPs items (32%), followed by polyethylene (14%), polyamide (10%), polystyrene (9%), polyethylene/polypropylene (7%), polyvinyl propionate and polyethylene terephthalate (6%), polypropylene (4%), polyacrylate/nylon/polypropylene and polyacrylate (3%), and polyester and polyvinyl chloride (3%).

## 4. Discussion

### 4.1. Fish Biometry

Biometric parameters (BP) of fish, such as total body length and weight, growth potential, and the overall condition of the fish, vary within the same species depending on various environmental, ecological, genetic, and geographical factors [148]. The length and weight of various fish species caught in different marine ecosystems, including the period of fishing, are summarized in Table 3. To improve the readability of the data presented in the table, a color scale is applied to express data collected in various periods.

*Clupea harengus* from the Atlantic, the Pacific, and from the North and Norwegian Seas are longer and heavier than the Baltic herring. Specimens from the North Sea are usually only slightly larger and approximately of the same weight as those from the BS. According to the current research, the average length and weight of the Baltic herring in the BS are 18.93 cm and 50.08 g, respectively. However, historical records indicate the occurrence of larger individuals, reaching total lengths of even up to 30 cm [119]. Research on the Baltic herring conducted between 1986 and 1996 showed an increase in body mass in the first few years (1986–1989). However, from the early 1990s, a decline in the body mass and length of herrings was observed in all areas of the BS. This body size decrease was related to a significant drop in the pelagic fish population and a consequent reduction in food availability [180]. Unfortunately, the average value of the total length of the Baltic herring recorded in the current study reveals that a decline in length is still observed. It could be caused by the impact of various environmental stressors, including pollution with MPs.

Analysis of the biometric characteristics of sprat, such as the average total length (11.36 cm) and weight (10.03 g), revealed changes in comparison to populations caught in other water bodies over recent decades. According to the literature, sprats from the Atlantic Ocean are larger than those found in the Mediterranean Sea, the Adriatic Sea, the BS, and particularly the Black Sea [155,156]; however, the sprats caught in the Black Sea were smaller than those caught in the BS [181].

Since the 1990s, a systematic reduction in the length and weight of adult cod in the BS has been observed [182]. According to reference records, adult cod in the BS are of a length of around 30 cm [164], and their weight is in the range of 300–1300 g [73,169], which is consistent with the data obtained in this study.

Summarizing the temporal variation in the total length and the total mass of herring, sprat, and cod, it could be concluded that specimens caught in the BS between the 1970s and 1990s were characterized by higher body length and weight values in comparison to contemporary individuals. The cause of this phenomenon is supported by various anthropogenic and environmental factors, such as pollution, overexploitation of large individuals, and warming seawaters due to climate change. The former causes a decrease in the average size of fish in the population [167,180,183,184,185,186], while the latter affects the metabolism of marine organisms, leading to changes in growth rates and nutrient availability [187,188]. However, assuming that entire shoals are equally exposed to some general categories of environmental stressors, such as deficiencies of food supply or increasing concentration of toxins in the BS, intraspecimen variation could be explained by specimen-related impact instead, such as individual contamination with MPs.

Flounders from the Atlantic are characterized by higher length than their counterparts from the BS, while specimens caught along the Portuguese and French coasts, the western coast of the Iberian Peninsula, or in the White Sea are generally smaller and lighter. Flounder populations from the North or Black Sea are characterized by a similar or higher length and weight in comparison to those caught in the BS. However, in areas bordering the BS (such as the Kattegat Strait), the total length of the flounder is comparable to Baltic populations, while specimens caught in various areas of the BS are characterized by similar length.

The lumpfish were characterized by a lower length and weight in comparison to individuals caught in the Atlantic [178], although specimens caught in different areas of the BS or Norwegian salmon farms were characterized by similar length and weight compared to individuals in the current study.

The long-spined bullhead caught in the BS in the framework of the current research were characterized by length and weight in the range of 17.80–26.80 cm and 80.73–650.35 g, respectively. Unfortunately, the biometric features of *Taurulus bubalis* are poorly studied, and according to only one reference, specimens caught in the waters of southwest England were shorter [179].

In general, fish caught in different basins were characterized by varying length and weight. This is primarily due to the geographical location of the particular marine ecosystem. Several environmental gradients, such as temperature, seasonality, and sunlight, which are interrelated and affect each other, differ according to latitude [189]. In particular, temperature-dependent processes control a fish’s life cycle [190], setting the dynamic of metabolism, nutrition, storage, and utilization of energy [191], including spawning migrations, growth, and reproduction [192,193]. Waters of the Atlantic Ocean are characterized by reasonable thermal stability, which provides more favorable living conditions for marine fauna. Additionally, the ocean enables access to a wide range of nutrient resources and diverse habitats [194]. In contrast to oceans, smaller ecosystems such as seas have limited surface area and volume, which decreases the availability of resources and may restrict fish population sizes. Fish populations from northern waters (e.g., the White Sea [195]) are smaller due to harsher climatic conditions and limited food availability, in comparison to fish from the central Atlantic.

Geographical isolation and the a limited gene flow between different populations impact biometric characteristics [196]. As an example, lumpfish caught in the BS and the Atlantic were characterized by various body lengths and weights, likely due to the separation of populations living in the Atlantic during the last glacial minimum [95].

### 4.2. MPs Contamination According to Species and Organs

#### 4.2.1. MPs Contamination According to Geographical Region

The percentage contribution of MPs found in fish caught in the BS was generally higher than that in fish caught in the North Sea (5.5%) [138], North Atlantic (28%) [197], and Central Atlantic (30%) [198], similar to that in fish from the Pacific (66%) [199], Ionian Sea (48%) [200], and Mediterranean Sea (58%) [201], and lower than that in fish from the Caspian Sea (80%) [202], Bering Sea (85%) [203], Yellow Sea (95.3%) [204], and Oman Sea (100%) [205]. Fish from semi-enclosed seas or those with limited connection to the ocean, such as the Mediterranean, Baltic, or Caspian Sea, are generally more contaminated with MPs in comparison to fish inhabiting oceans or open seas. Semi-enclosed seas, such as the BS, are characterized by limited water exchange with the global ocean [206]. Due to the limited water exchange, the sea is more exposed to the accumulation of pollutants, MPs in particular, and is characterized by lower retention. Its resistance is additionally strongly impacted by local sources of emissions, such as cities, ports, river transport, waste, and shipping.

Although overall pollution with MPs increases over time, a general comparison of contamination with MPs in fish in the BS with that in fish caught in other regions indicates a certain variability. Other studies on fish from the BS indicate a lower abundance of MP items. The contribution of fish contaminated with MPs caught along the Swedish coast was 36.5% [104] and 50.4% [105]; along the Danish coast, it was 11 –21% [106]; along the Polish coast, it was 12.7–14.8% [73]; while in the Bornholm Basin, the reported contribution was 5.5% [138]–20% [75]. Since, as highlighted above, the risk of a failure to classify items as MPs was minimized, the discrepancies in these results may be attributed to the passage of time between the different research studies, the use of different sampling methods and processing procedures, seasonal and/or species-specific differences in feeding biology [75], and variations in the interpretation of findings [73]. For example, Białowąs et al. [73] excluded synthetic fibers suspected of being of airborne origin. In the current research, none of the MPs were found in blanks, minimizing the misclassification of items of airborne origin. All items were found in fish, and even though fibers may enter into the water from the air, they can still be swallowed by fish. In our opinion, the comparison of both sets of results obtained via the use of different methodological protocols could be ambiguous, and this is why the current results are of great scientific soundness.

#### 4.2.2. MPs in Marine Fish Species

Based on differences in the percentage contribution of MPs (Table 2), it has been revealed that pelagic fish, such as the sprat, the cod, and the herring, were generally characterized by a slightly lower abundance of items in comparison to demersal fish, such as the flounder and the lumpfish. This could be because the herring and the sprat feed mostly in the water column, away from the bottom. Most often, they feed in the upper water layers [207]; however, the cod also inhabits deeper waters [208]. The herring and the sprat travel over large areas for food and avoid bottom sediments, where MPs often accumulate in higher amounts due to biofouling [209]. On the other hand, the flounder and the lumpfish feed mostly in the lower water layers [210], which increases their exposure to MPs immobilized in the bottom sediments. Higher exposure of demersal fish to MPs was also confirmed by previous studies in the Thames Estuary, where benthic fish (*European flounder*) were contaminated with MPs at a rate of 75%. This was a significantly higher rate than that found in pelagic fish (*European smelt*), where only 20% of individuals were contaminated with MPs [211]. Similar results were reported in China [212], the Beibu Gulf in the South China Sea [213], and the Gulf of Mannar [214], where demersal fish ingested more MPs than pelagic fish.

#### 4.2.3. Accumulation of MPs in Organs

The presence of MPs in the gills has been reported in previous studies on different fish species [213,215]. Similarly, as in the current study, a higher or comparable accumulation of items in the gills in comparison to the intestines or the entire gastrointestinal tract has been observed [216,217]. The gills provide the first barrier protecting against stress factors and therefore may retain more MP items through passive filtration as water flows through the gill filaments [204]. MPs can easily adhere to the surface of the gills, and they can also penetrate blood vessels and cell membranes if the particle size is small enough, thereby impairing respiration in fish [218].

The share of MPs in the gastrointestinal tract was in agreement with the expectations and has been confirmed by previous research on various fish species from Southern Puglia [219], Northern Thailand [220], the Oman Sea [205], the Black Sea [221], and the Atlantic Ocean [222]. MPs enter the gastrointestinal tracts of fish through various pathways, such as by consuming contaminated prey or mistaking them for food [104,222]. Therefore, the gastrointestinal tract, like the gills, is one of the primary ways of MPs contamination [223]. As presented above (Table 2), these conclusions are supported by statistical analyses, which did not confirm a significant difference in MPs abundance between the gastrointestinal tract and the gills.

The current study revealed that MPs items could be translocated to the liver from other organs. Although the translocation of MPs items with a size between 200 and 600 μm to the liver of *Mugil cephalus*, *Engraulis encrasicolus*, and *Sparus aurata* was also previously confirmed [224,225,226], the observed translocation of such large particles (even up to 250 μm) is difficult to explain based only on current knowledge of MPs translocation pathways. This is why intensive research concerning possible translocation mechanisms is still being carried out. As a result, two main translocation routes have been proposed, including transcellular and paracellular routes [224]. The transcellular route involves absorption through the microvillus border into the bloodstream. In mammals, transcellular uptake occurs mainly through M cells in Peyer’s patches and the gut-associated lymphoid tissue (GALT). Instead of GALT, fish possess lymphoid cells and macrophages [224]. Recent studies on salmonids [227] and *D. rerio* [228] have identified specialized enterocytes with M cell-like activity in the posterior part of the midgut, which may be responsible for the uptake and transfer of MPs to closely associated macrophages capable of phagocytosis [228]. The paracellular transport pathway involves the passage of particles through tight junctions between cells, and in cases of inflammation, it may be facilitated by tissue damage [229] and the agglomeration of smaller MPs or even nanoplastics [230].

#### 4.2.4. Physical and Chemical Characterization of MPs

MPs items in the size range of 0.11–0.5 mm and 1.01–5 mm dominated in fish organs. Earlier studies have also confirmed that MPs in the size range of 0.1–0.5 mm are the dominant size identified in fish samples, including records from the Black Sea [231], Northern Ionian Sea [200], the Mediterranean Sea [232], and the Bay of Bengal [233]. The dominance of MPs in the size range of 0.1–0.5 mm and 1.01–5 mm indicates that fish are unable to distinguish them from their natural prey, as both fall within the same size range [234]. Current results have shown that the liver contains smaller items in comparison to the gills and the gastrointestinal tract, which is consistent with other scientific reports [31,205]. Larger MPs are easily filtered by the fish gills, while smaller items initially accumulate in the gut and are then transferred to the liver [235].

The most common MPs identified in fish organs were blue, black, and red. The color of MPs could potentially increase their bioavailability due to their resemblance to prey items, especially to visual raptorial species [236,237]. Similarly, Białowąs et al. [73] found a predominantly analogous color scheme within the gastrointestinal tract of herring and cod caught in the southern BS. Additionally, the trend of blue MPs dominating in fish organs has also been observed in other marine ecosystems worldwide [238,239].

Fibers were the most common plastic shape detected in fish samples. Similar percentage contributions of fibers were found in fish from the Red Sea [240], the China Sea [212], and the Mediterranean Sea [241]. Fibers can be mistaken for food by fish due to their morphological resemblance to natural items such as algal filaments, oligochaetes, nematodes, amphipods, and polychaetes [242].

The most popular types of polymers were cellophane (32%), polyethylene (14%), polyamide (10%), and polystyrene (9%). The current study is consistent with findings from other studies regarding MPs items in fish from marine ecosystems [100,212]. These polymer types are commonly used for food, clothing, and other packaging applications [243]. Of the 359 million tons of plastics manufactured in 2018, over 40% were intended for packaging, a commonly short-lived, single-use application [244]. The high contribution of cellophane in fish organs may result from the fact that it was one of the first polymers used for food packaging. Additionally, it can be coated with other polymers to improve its heat and water resistance, as well as its adhesive properties for use in tapes, labels, photographic film, and other applications [245]. Polyamide and polystyrene are widely used in the textile industry and packaging production, making them significant sources of MPs in marine environments [246]. Due to degradation processes, these materials break down into MPs items with various shapes, particularly fibers, making them accessible to aquatic organisms [247].

### 4.3. Fish Condition Status

#### 4.3.1. K Factor

Several studies confirmed the strong positive relationship between the K factor and the total lipid content of fish [248,249] and assigned the K factor as a simple proxy of energy reserves in the fish body. Table 4 summarizes a literature review on the K factor values of six marine fish species inhabiting various water reservoirs. The available literature lacks criteria for assessing the K factor between various species. Based on the unified results (Figure 3), it was observed that fish caught in different marine areas were characterized by different K factor values. Fish caught in the eastern Atlantic were generally characterized by better health conditions and higher K factor values in comparison to fish from the north and northwestern Atlantic. Species caught in the North Sea and neighboring Atlantic basins were characterized by good to moderate health conditions. This is because there is unlimited water exchange with the Atlantic in the North Sea, which creates more favorable environmental conditions for marine organisms. Fish from the BS were characterized by lower K factor values in comparison to individuals from the Atlantic and North Sea, but in this case, there was also variation between basins. In comparison to individuals from the central and northern parts of the BS, fish from the areas bordering the North Sea were in better health. It was also observed that some populations from the southern and southeastern BS were characterized by good to moderate health conditions.

The health condition of *Clupea harengus* examined in this study was assessed as poor. Herrings from the Atlantic, North Sea, and White Sea were characterized by higher and mutually comparable K factor values. Herring caught in the northern and eastern parts of the BS were characterized by lower K-values in comparison to those from the southwestern region, while fish from the central part of the BS were characterized by a similar K factor in comparison to the northeastern region. Once again, assuming an equality of exposition of entire shoals to xenobiotics released to marine waters, as well as a comparable impact of food supply level, the observed intraspecimen variation in the K index could be associated with an impact of specimen-related stressors, such as individual contamination with MPs. This is why the multivariate assessment based on the most abundant species, such as the Baltic herring, was desired. Its outcome is discussed in Section 4.4.

*Sprattus sprattus* caught in the central part of the BS were characterized by lower or comparable K factor values, in comparison to individuals from other European regions or the Black Sea. On the contrary, sprats from the North Sea were characterized by a higher K factor in comparison to the BS, indicating better health conditions. In comparison to other studies conducted in the BS, sprats from the Gulf of Gdansk and Bothnian Bay were characterized by lower K factor values.

The Baltic cod was characterized by comparable or lower K factor values than cod from the Atlantic [127,128,129,130], the North Sea [131], and the Irish Sea [132]. The comparison of K factor values between specimens caught in different subbasins of the BS indicates spatial variation. Cod from the Gulf of Gdańsk [166] and the Bornholm Basin [136] were characterized by higher K factor values, while specimens from the Kattegat Strait [138] by lower values in comparison to cod caught in the central BS (Table 4).

Flounders caught in the central part of the BS were characterized by slightly higher K factor values, in comparison to individuals from other regions, such as the western coast of France, the English Channel, the Atlantic coast of Portugal, and the Mersey and Dee estuary, Irish Sea. Flounders caught in various areas of the BS were also characterized by slightly different K factor values, revealing similarities in the spatial variation in the K factor determined for cod (Table 4).

The highest K factor was recorded for *Cyclopterus lumpus*. Previous studies have been focused exclusively on farmed lumpfish in Norway, where wild lumpfish are extensively used as a cleaner fish in the salmon aquaculture industry. A range of K factors was reported for farmed lumpfish: 2.6–4.2 in 2018, 4.1–5.1 in 2019, and 4.1–4.7 in 2020 [251,252,253]. Since the range of K factor values is comparable to those obtained in the present study, a consistency between the health statuses of wild and farmed populations in terms of condition is confirmed. According to the criteria proposed by Imsland et al. [254] for the lumpfish, K factor values between 4.5 and 5.5 are an indicator of good health, values between 3.5 and 4.5 reflect a moderate condition, while K values ranging from 3 to 3.5 indicate poor health. Fish with K values below 3 are classified as emaciated and in very poor health. In our study, the lumpfish caught as bycatch in the BS exhibited K factor values within the range 3.51–4.5, which indicates moderate health conditions. These results provide a valuable contribution for the understanding of lumpfish health conditions in wild populations, which have not previously been the subject of detailed investigations within their natural habitat.

The K factor was also analyzed for six individuals of *Taurulus Bubalis*, revealing an average of 1.97. According to the available literature, there is only one relevant study, conducted by Barrett et al. [250], who examined the condition of the long-spined bullhead. However, it should be noted that Barrett et al. [250] applied a modified formula to calculate the K factor, and because of that, direct comparison of the results is problematic. Due to the lack of established criteria for evaluating the K factor of this species, it is difficult to conclusively assess the condition of the individuals analyzed in the current research. The K factor values obtained ranged from 1.14 to 3.48, which complicates a clear assessment of their health status; however, according to Barrett et al. [250], we assess the welfare of the long-spined bullhead as moderate-to-good.

Based on the summarized K factor values, fish from the Atlantic were characterized by higher K factor values [109,139,143], likely due to easy access to food, such as plankton and small fish [255], and favorable conditions like high salinity [256]. Food supply and salinity support better health and nutrition for marine species. High K factor values reported for fish from the North Sea are also related to nutrient availability and the influence of Atlantic saline waters. In the case of the BS, pelagic fish such as sprat caught in the colder waters of the Bothian Bay were characterized by lower K factor values. This is likely due to colder water and limited food supply [257]. Fish condition in colder climates often depends on water temperature and food availability, while fish from warmer, nutrient-rich (eutrophic) areas tend to be characterized by higher K factor values [258]. Cod from the Kattegat were characterized by higher K factor values in comparison to those in other parts of the BS, likely due to warmer water temperatures and greater food supply in that region. The Baltic flounders adapted to the unfavorable environmental conditions and pollution in BS, as they were characterized by a higher K factor in comparison to individuals from other European ecosystems. This suggests physiological adaptations that allow them to survive and even thrive despite increased pollution levels.

#### 4.3.2. HSI

The HSI is an important indicator of fish condition status [259], including their metabolic health [260,261]. The liver, as a key organ responsible for detoxifying the organism, plays a central role in neutralizing toxins and metabolizing chemicals present in water, such as heavy metals and organic pollutants [262]. Fish characterized by low HSI values may be more susceptible to the adverse effects of pollution, which could lead to deteriorated physiological conditions, reduced disease resistance, and lower fertility [263]. Several studies have demonstrated links between environmental pollution levels and diseases in fish [263]. For example, Vethaak and Jol [264] reported associations between sediment contamination by polycyclic aromatic hydrocarbons and polychlorinated biphenyls and liver diseases in sea fish during experiments.

Three of the five species examined were characterized by similar HSI values (0.02–0.03), indicating a relatively uniform physiological liver condition. The long-spined bullhead and the lumpfish were characterized by a higher HSI, in the range of 0.06–0.09. According to our knowledge, there are no studies assessing the HSI for the wild long-spined bullhead and lumpfish. The only available studies concern lumpfish caught in Norwegian salmon farms, which were characterized by lower HSI values [253] in comparison to those observed in this study. Based on the literature, flounder, cod, and herring caught in other marine ecosystems and the BS in earlier studies were characterized by similar HSI values in comparison to current data. Flounder caught in the estuary in Portugal were characterized by HSI values ranging from 0.015 to 0.025 [143], while the population from the Irish Sea had values equal to 0.015 [144]. Cod caught in the Atlantic were characterized by HSI values in the range of 0.03–0.12 [133]. The current result of HSI values for the flounder matches corresponding HSI values for individuals caught in different areas of the BS, such as the Gulf of Gdańsk [171] and the open BS [145], which were characterized by values in ranges of 0.015–0.022 and 0.017–0.026, respectively. Research by Lang et al. [265] also suggests that flounder from the Gulf of Gdańsk were less affected by diseases than those from Kvädöfjärden and the Lithuanian coast, highlighting the complexity of connections between pollution and fish health. The HSI values calculated for herring and cod caught in the Arkona Basin [266] and the Bornholm Basin [136] were also consistent with the current results. Relatively uniform HSI values suggest that environmental differences, such as water temperature, salinity, and pollution levels, do not significantly impact the HSI. The HSI and hence the values reported for the species inhabiting the various sub-basins of the BS remain relatively stable. This may indicate adaptive physiological mechanisms that allow fish to cope with challenging environmental conditions.

#### 4.3.3. GILSI

Fish in marine ecosystems are exposed to various stressors, triggering a significant impact on the functioning of their organs, particularly the gills [267,268]. Due to their large surface area and direct contact with water, fish gills can be impacted by xenobiotics, which can disrupt their function even at the lowest concentrations. It is well known that exposure of fish to toxic chemicals can lead to various histopathological lesions in vital organs, including hyperplasia and hypertrophy [269,270]. Histopathological changes in fish gills have previously been considered valuable biomarkers of aquatic ecosystem stressors [36,271]. However, to our knowledge, no studies have examined gill mass in individual species. The ratio of gill mass to total fish mass was determined at a comparable level for all fish (0.03–0.04). This may indicate no interspecimen variation. Due to the lack of studies on gill mass in the literature, a comparison of results is challenging. Therefore, the data presented in the current study are novel and may contribute to a better understanding of future research.

#### 4.3.4. GITI

The gastrointestinal tract plays a key role in the physiological processes of fish, and its importance increases in the context of exposure to chemical pollutants present in aquatic environments [272]. Pollutants ingested by fish can pass from the gastrointestinal tract into the bloodstream and from there spread to other internal organs such as the liver, the kidneys, or the muscles [273]. For this reason, the gastrointestinal tract represents an important point of contact between the fish’s body and potential xenobiotics, which may affect the overall health of the fish and the functioning of organ systems. A lack of interspecimen differentiation was also observed in the ratio of gastrointestinal tract mass to total fish mass (0.04–0.11). The current results serve as a valuable reference point, but further research is needed to better understand the relationship between the gastrointestinal tract mass and environmental factors. To our knowledge, previous research has not focused on measuring the ratio of gastrointestinal tract mass to total fish mass in the context of xenobiotic exposure or other environmental stressors. However, studies have been concerned with histological abnormalities associated with heavy metal exposure and their accumulation in the digestive systems of fish [274,275]. The current results are novel and provide a foundation for future, more detailed studies. Understanding the impact of environmental stressors on the fish digestive system can provide important insights for the protection of aquatic ecosystems and the health of fish populations.

### 4.4. Multidimensional Analysis Involving Baltic Herring

As mentioned above, the relationship between fish well-being indices and MPs abundance was evaluated by PCA. The majority of fish samples are located within the range of −0.5 to +0.5 value of the factor scores and create only slightly spread groups in the center of the PC coordinate system, with only two samples that are significantly far away from the others. The single sample located on the left-hand side of the biplot was collected in 103 fishing zones on the 26th of February, 2022, and was characterized by negative factor scores along PC1, which was contributed by high values of HSI, GITI, and GILSI and a low abundance of MPs. In this case, the significant distance from the origin of the coordinate system was caused by high values of the GITI due to the presence of significant amounts of undigested food in the gastrointestinal tract. The weight of the tract of this individual was equal to 7.1 g, while the average calculated for all herrings was equal to 1.8 g. On the contrary, the single sample located on the top of the biplot was collected in 135 fishing zones on the 21st of October, 2023. It was characterized by a positive factor score value along PC2, which corresponds to the highest observed K factor value. In this case, the individual was characterized by a small basic length (13.9 cm, average 16.2 cm) and higher mass, in comparison to other herrings, which was equal to 74.4 g (average 50.2 g). Apart from these two different individuals, the location of samples according to fishing zones indicates some relation between the abundance of MPs and the well-being indices. Since the higher the factor score, the higher the impact of the factor (in terms of the value of the given variable contributed to the factor), generally, better well-being indices corresponding to a lower abundance of MPs were confirmed. Moreover, some spatial variation between well-being indices of herrings caught in various fishing zones was also observed. The majority of herrings caught in fishing zone 108 on the 15th of November 2021, as well as those caught in fishing zone 103 on the 26th of February 2022, were characterized by positive factor scores along PC1 and slightly negative scores along PC2, respectively. This indicates that these individuals were characterized by decreased K factor values due to an increased abundance of MPs. This generally fits with the observation that the area of the southern BS, the coastal one in particular, is more polluted with MPs [206], and hence, herrings are more exposed. Accidental or deliberate ingestion of MPs negatively impacts the overall well-being of shoals of herrings. On the contrary, herrings caught in fishing zone 135 on the 24th of April, 2023, and 21st of October 2023, as well as in fishing zone 129 on the 24th of April 2023, were instead characterized by negative scores along PC1 and positive ones along PC2, indicating a negligible negative impact of MPs on the calculated well-being indices. As a logical consequence, it could be concluded that these herrings were characterized by higher values of condition factors due to a decreasing abundance of MPs. Multidimensional analysis successfully revealed that the condition of shoals of Baltic herrings could be assessed based on condition indices according to fishing zones and, in general, confirms that there is a link between pollution of MPs and fish health. Shoals of Baltic herrings from the Baltic Proper are less impacted by MPs and hence their well-being indices are characterized by higher values, indicating a better condition of individuals than shoals feeding in the southern Baltic, which seems to be more polluted by MPs due to surface release. The findings of the current study are consistent with other results acquired by analyses of wild fish from the Iberian Peninsula [276], the Northeastern Atlantic Ocean [277], the Bay of Bengal [278], and the Mediterranean Sea [279], as well as with condition index values computed for other species in controlled experiments [280,281]. In all references presented above, the lower the K factor, the greater the contamination with MPs confirmed in fish. Although it is difficult to present an unambiguous explanation concerning the cause and the effect in this case, we hypothesize that the presence of MPs in the gastrointestinal tract may reduce food intake, leading to a negative impact on feeding activity and body weight gain [282,283]. Some authors highlight this effect, particularly in the case of prolonged MPs retention [280,284]. Moreover, a decrease in body weight and in general well-being of fish could also be associated with the release of xenobiotics from MPs, which was also confirmed by others [219,285]. Nevertheless, it needs to be emphasized that the multidimensional approach revealed significant insight into this topic, and more studies in these fields are needed.

## 5. Conclusions

The current study discussed biometric features such as length and weight, as well as condition factors (K, HSI) for herring and bycatch species from the BS. For two species, i.e., the wild lumpfish and the long-spined bullhead populations, health condition indices were measured for the first time. In addition, our study proposed two new indices, GILSI and GITI, to improve fish health assessment methods.

The overall conclusion reveals that in recent decades, a trend of decreasing fish size has been observed in the BS. This phenomenon is influenced by various anthropogenic and environmental factors, such as the overexploitation of large individuals, warming seawaters due to climate change, and pollution. Fish caught in different basins, such as the Atlantic and Pacific Oceans, the North Sea, and the Norwegian Sea, are characterized by higher values of length and weight. This is primarily due to the geographical location of the particular marine ecosystem. The variation in biometric features, such as the length and weight of the fish, makes it possible to assess their health condition, as these parameters influence the K factor value. Studies have shown that fish from the Atlantic and North Sea have higher K factor values. This is because there is unlimited water exchange with the Atlantic in the North Sea, which creates more favorable environmental conditions for marine organisms. Therefore, the assessment of the biometric features of fish is an important tool for monitoring their health and the impact of local hydrological conditions on fish populations in different marine areas. A similar phenomenon was observed for fish from the Baltic Sea, where individuals caught in the northern areas of the basin were characterized by a lower K factor value in comparison to fish from areas adjacent to North Sea waters. This suggests that access to more abundant ocean waters may have a positive effect on fish conditions. To allow for a comparison of the K factor between species, this study has proposed its normalization, scaling the K values to a universal range from 0 to 1. The introduction of a normalized scale may better highlight differences in interspecies fish health conditions and may provide a new tool for future research. The relatively uniform HSI values in the BS and other marine basins suggest that environmental differences such as water temperature, salinity, and pollution levels do not have a significant impact on the condition of fish, including their metabolic health. The ratio of the gill (GILSI) and the gastrointestinal tract (GITI) mass to total fish mass was determined at a comparable level for all fish. However, to our knowledge, no studies have examined the gill mass and the gastrointestinal tract mass in individual species. Therefore, the data presented in the current study are novel and may contribute to a better understanding in future research. MPs items were identified in all species, with the highest contribution in lumpfish and the lowest in sprats. The highest number of plastic particles was identified in the gills, followed by the gastrointestinal tract, indicating that the MPs present in the water are trapped in the gill arches through filtration and respiration and are also ingested accidentally with food. A possible translocation of MPs to the liver was also considered and observed. The general abundance of MPs in the studied organs of BS fish was similar to that in other studies. Multidimensional analysis successfully revealed that shoals of Baltic herrings could be assessed based on condition indices according to fishing zones and, in general, confirmed that there is a link between MPs pollution and fish health condition. The analysis showed that Baltic herring that were less contaminated with MPs were characterized by higher K values, indicating better health in comparison to individuals more contaminated with MPs, which is consistent with other studies. The fish health condition factors and proposed indices can be considered important for monitoring ecological risks for fish exposed to MPs pollution and other environmental stressors. The morphological and chemical features of MPs found in marine fish organs were well aligned with the features of plastic items found in analogous samples from other aquatic reservoirs. This indicates that the level of anthropogenic pressure, and the related release of MPs, is similar across various locations according to size and chemical composition.

## Figures and Tables

**Table 1:** Basic statistics of morphometric characteristics of fish and their health condition indices.

Species (Latin Name, Feeding Features, Habitat Zone)	Variable [Unit]	N	Mean	Median	Minimum	Maximum	S.D.
Baltic herring (*Clupea harengus*, planktivore, pelagic)	Total mass [g]	128	50.08	45.87	16.87	108.04	15.71
	Liver mass [g]	125	0.75	0.61	0.12	2.51	0.45
	GIT mass [g]	126	1.86	1.70	0.04	9.73	1.18
	Gills mass [g]	126	1.61	1.67	0.51	2.98	0.48
	Total length [cm]	128	18.93	18.70	13.50	28.30	2.48
	Basic length [cm]	117	16.16	15.70	11.40	23.50	2.39
	K factor	128	0.75	0.71	0.39	2.04	0.22
	HSI	125	0.02	0.01	0.00	0.06	0.01
	GILSI	126	0.03	0.03	0.01	0.08	0.01
	GITI	126	0.04	0.04	0.00	0.17	0.02
flounder (*Platichthys flesus*, carnivore, demersal)	Total mass [g]	46	217.13	189.12	88.30	580.74	126.02
	Liver mass [g]	46	3.76	3.53	1.02	8.77	1.95
	GIT mass [g]	46	9.19	7.97	2.41	25.11	5.45
	Gills mass [g]	46	6.23	5.35	3.05	15.80	2.97
	Total length [cm]	46	25.52	24.95	17.10	34.30	4.14
	Basic length [cm]	46	20.95	20.20	15.20	30.80	3.61
	K factor	46	1.29	1.11	0.57	3.71	0.59
	HSI	46	0.02	0.02	0.01	0.05	0.01
	GILSI	46	0.03	0.03	0.02	0.04	0.01
	GITI	46	0.05	0.04	0.01	0.20	0.03
Baltic cod (*Gadus morhua*, omnivore, demersal/pelagic)	Total mass [g]	30	284.64	248.47	78.14	750.12	170.69
	Liver mass [g]	27	8.22	6.39	1.74	18.83	5.02
	GIT mass [g]	27	10.86	7.88	1.47	45.96	8.36
	Gills mass [g]	27	9.21	8.14	3.64	19.53	3.79
	Total length [cm]	30	29.98	29.40	20.80	41.80	4.22
	Basic length [cm]	30	24.74	24.80	16.80	32.10	3.69
	K factor	30	0.98	1.00	0.37	1.70	0.31
	HSI	27	0.03	0.03	0.01	0.11	0.02
	GILSI	27	0.04	0.04	0.02	0.06	0.01
	GITI	27	0.04	0.04	0.00	0.09	0.02
lumpfish (*Cyclopterus lumpus*, carnivore, demersal)	Total mass [g]	19	165.54	158.87	110.06	271.70	39.04
	Liver mass [g]	15	13.93	14.32	10.23	20.46	2.49
	GIT mass [g]	15	18.55	19.67	10.04	30.58	5.16
	Gills mass [g]	15	4.60	4.58	2.86	7.73	1.30
	Total length [cm]	19	15.49	15.50	13.90	18.80	1.25
	Basic length [cm]	19	12.94	12.80	11.80	15.10	0.89
	K factor	19	4.41	4.22	3.90	5.94	0.49
	HSI	15	0.09	0.09	0.06	0.11	0.02
	GILSI	15	0.03	0.03	0.02	0.04	0.00
	GITI	15	0.11	0.11	0.08	0.14	0.02
long-spined bullhead (*Taurulus bubalis*, carnivore, demersal)	Total mass [g]	6	284.61	221.92	80.73	650.35	208.58
	Liver mass [g]	6	12.49	9.80	5.40	21.32	6.46
	GIT mass [g]	6	36.02	23.79	5.84	120.42	42.74
	Gills mass [g]	6	9.23	9.20	3.45	13.03	3.56
	Total length [cm]	6	23.33	23.85	17.80	26.80	3.06
	Basic length [cm]	6	18.93	19.60	15.20	20.80	2.03
	K factor	6	1.97	1.62	1.14	3.38	0.86
	HSI	6	0.06	0.05	0.02	0.14	0.04
	GILSI	6	0.04	0.05	0.02	0.06	0.02
	GITI	6	0.11	0.12	0.04	0.19	0.06
sprat (*Sprattus sprattus*, planktivore, pelagic)	Total mass [g]	28	10.03	9.81	6.09	15.34	1.95
	Total length [cm]	28	11.36	11.40	10.10	13.60	0.92
	Basic length [cm]	28	9.58	9.50	8.30	11.50	0.76
	K factor	28	0.69	0.66	0.48	1.13	0.12

Note: K factor—Fulton’s condition factor; HSI—hepatosomatic index; GILSI—the ratio of gill mass to the total body mass of fish; GITI—the ratio of gastrointestinal tract mass to the total body mass of fish; S.D.—standard deviation.

**Table 2:** Percentage share of fish with MPs identified, the distribution of plastic items according to the type of fish organ, and statistical assessment of differences between MPs concentrations across mutual combinations of fish species.

Species	N_MPs_/N_all_	Share of Fish with MPs	MPs Found in Organs (Items), (%)			Total
			Liver	Gills	Gastrointestinal Tract	
Baltic herring	80/128	63%	33(15%)	99 (45%)	89 (40%)	221
Baltic cod	17/30	57%	9 (18%)	25 (49%)	17 (33%)	51
flounder	28/46	61%	19 (16%)	47 (39%)	54 (45%)	120
lumpfish	15/19	79%	10 (16%)	39 (63%)	13 (21%)	62
long-spined bullhead	4/6	67%	1 (12%)	4 (50%)	3 (38%)	8
sprat	6/28	21%	n.c.	n.c.	n.c.	9
total	150/257	59%	72 (15%)	214 (45%)	176 (37%)	471

Note: N_MPs_—number of specimens with MPs identified in their organs; N_all_—total number of specimens; n.c.—not counted. Percentage share of MPs per organ does not equal 100% in sum since organs were not investigated in sprat.

**Table 3:** The length and weight of various fish species caught in different marine ecosystems worldwide according to the period of fishing.

Species	Location		Length					Mass										References
Baltic herring			10–16	17–22	23–28	29–34	>35	10–50	50–100	101–150	151–200	201–250	251–300	301–350	351–400	401–450	>450	
	BS	southern BS																current study
		southern BS																[119]
		central BS																[118]
		northern BS																[113]
		Estonian coast																[149]
	Northeast Pacific																	[150]
														
	Northwest Atlantic																	
	Northeast Atlantic																	
														
	North Atlantic																	[151]
														
														
	Norwegian Sea																	[150]
																		[152]
	North Sea																	[109]
																		[111]
																		[138]
sprat			5–7	7.1–9	9.1–11	11.1–13	>13	1–2	2.1–4	4.1–6	6.1–8	8.1–10	10.1–12	12.1–14	14.1–16	16.1–18	>18	
	BS	southern BS																current study
		Baltic Proper																[153]
														
														
																		[154]
	Atlantic																	[155]
																		[156]
	Mediterranean Sea																	[157]
	Adriatic Sea																	[158]
	Black Sea																	[159]
																		[160]
																		[161]
																		[162]
	North Sea																	[163]
Baltic cod			20–30	31–40	41–50	51–60	>60	200–300	301–400	401–500	501–600	601–700	701–800	801–900	901–1000	1000–1200	>1200	
	BS	southern BS																current study
		Bornholm coast																[164]
		Baltic Proper																[165]
		Bornholm Basin/Gdansk Deep																[166]
		southern BS																[167]
		Bornholm Basin																[168]
		southern BS																[73]
		eastern BS																[169]
														
flounder			15–20	21–25	26–30	31–35	>36	200–300	301–400	401–500	501–600	601–700	701–800	801–900	901–1000	1001–1200	>1200	
	BS	southern BS																current study
		Hanö Bight, Gotland coast																[147]
		all BS																[170]
		Polish coast																[171]
		Polish coast																[172]
		northern BS																[173]
	Atlantic Ocean																	[172]
																		[140]
																		[143]
	North Sea																	[147]
	Black Sea																	[174]
lumpfish			15–20	21–30	31–40	41–45	>45	100–200	201–300	301–400	401–500	501–600	601–700	701–800	801–900	901–1000	>1000	
	BS	southern BS																current study
		central, southern BS																[175]
	Norwegian fish farms																	[176,177]
	Western Atlantic																	[178]
	Eastern Atlantic																	
	English Channel																	
long-spined bullhead			5–10	11–15	16–20	21–25	>25	50–100	101–200	201–300	301–400	401–500	501–600	601–700	701–800	801–900	>900	
	BS																	current study
	Southern England																	[179]
period	mass	length
1960–1979		
1980–1999		
2000–2009		
2010–2019		
2020–2024		

**Table 4:** Summary of literature review on K factor values of the Baltic herring, the sprat, the Baltic cod, the flounders, the long-spined bullhead, and the lumpfish.

Species	Location	K Factor			References
Baltic herring		<0.9	0.91–1.1	>1.1	
	central BS				current study; [114,116,119]
	northern and eastern BS				[114,116]
					[115]
	southwest BS				[120]
	Atlantic Ocean				[109]
					[110]
					[111]
	North Sea				[112]
sprat	BS				current study; [125,126]
	Black Sea				[121,122]
	North Sea				[123]
					[124]
Baltic cod	BS				current study
					[136]
					[137]
					[138]
	Atlantic				[127,128,129,130]
					[133,134,135]
	North Sea				[131]
					[132]
	Irish Sea				[127]
flounders	BS				current study
					[145]
					[146]
					[138]
					[147]
	Atlantic				[139]
					[140]
					[141,142]
					[143]
	Irish Sea				[144]
long-spined bullhead	BS				current study
	Atlantic				[250]
lumpfish		3–3.5	3.51–4.5	4.51–5.5	
	BS				current study
	North Sea (salmon farms)				[251]
					[252,253]

## Data Availability

The data presented in this study are available on request from the corresponding author. The data are not publicly available due to the fact they will be a part of a Ph.D. thesis.

## Supplementary Materials

The following supporting information can be downloaded at: https://www.mdpi.com/article/10.3390/ani15162381/s1. Table S1. Spearman’s correlation coefficient values between total length and total mass of the fish according to species.

## References

[1] Holmlund C., Hammer M. (1999). Ecological ecosystem services generated by fish populations. Ecol. Econ..

[2] Sterner R.W., Elser J.J. (2002). Stoichiometry and homeostasis. Ecological Stoichiometry: The Biology of Elements from Molecules to the Biosphere.

[3] Traugott M., Thalinger B., Wallinger C., Sint D. (2021). Fish as predators and prey: DNA-based assessment of their role in food webs. J. Fish. Biol..

[4] Schiettekatte N.M.D., Casey J.M., Brandl S.J., Mercière A., Degregori S., Burkepile D., Van Wert J.C., Ghilardi M., Villéger S., Parravicini V. (2023). The role of fish feces for nutrient cycling on coral reefs. Oikos.

[5] Whitfield A.K. (1996). Fishes and the environmental status of South African estuaries. Fish. Manag. Ecol..

[6] Zhang Y., Wang X.N., Ding H.Y., Dai Y., Ding S., Gao X. (2019). Threshold responses in the taxonomic and functional structure of fish assemblages to land use and water quality: A case study from the Taizi River. Water.

[7] Gebremedhin S., Bruneel S., Getahun A., Anteneh W., Goethals P. (2021). Scientific methods to understand fish population dynamics and support sustainable fisheries management. Water.

[8] Finegold C., Wramner P., Cullberg M., Ackefors H. (2009). The importance of fisheries and aquaculture to development. Fisheries, Sustainability and Development.

[9] Bosch A.C., O’Neill B., Sigge G.O., Kerwath S.E., Hoffman L.C. (2016). Heavy metals in marine fish meat and consumer health: A review. J. Sci. Food Agric..

[10] Mostofa K., Liu C.Q., Vione D., Gao K., Ogawa H. (2013). Sources, factors, mechanisms and possible solutions to pollutants in marine ecosystems. Environ. Pollut..

[11] Bashir I., Lone F.A., Bhat R.A., Mir S.A., Dar Z.A., Dar S.A., Hakeem K., Bhat R., Qadri H. (2020). Concerns and threats of contamination on aquatic ecosystems. Bioremediation and Biotechnology.

[12] Gissi E., Manea E., Mazaris A.D., Fraschetti S., Almpanidou V., Bevilacqua S., Coll M., Guarnieri G., Lloret-Lloret E., Pascual M. (2021). A review of the combined effects of climate change and other local human stressors on the marine environment. Sci. Total Environ..

[13] Malik D.S., Sharma A.K., Thakur S., Sharma M. (2020). A review on impact of water pollution on freshwater fish species and their aquatic environment. Advances in Environmental Pollution Management: Wastewater Impacts and Treatment Technologies.

[14] Dijoux S., Boukal D.S. (2021). Community structure and collapses in multichannel food webs: Role of consumer body sizes and mesohabitat productivities. Ecol. Lett..

[15] Laufkötter C.A.O., Zscheischler J.A.O., Frölicher T.A.O. (2020). High-impact marine heatwaves attributable to human-induced global warming. Science.

[16] Eriksen M., Lebreton L.C.M., Carson H.S., Thiel M., Moore C.J., Borerro J.C., Galgani F., Ryan P.G., Reisser J. (2014). Plastic pollution in the world’s oceans: More than 5 trillion plastic pieces weighing over 250,000 tons afloat at sea. PLoS ONE.

[17] Galafassi S., Sighicelli M., Pusceddu A., Bettinetti R., Cau A., Temperini M.E., Gillibert R., Ortolani M., Pietrelli L., Zaupa S. (2021). Microplastic pollution in perch (*Perca fluviatilis*, Linnaeus 1758) from Italian south-alpine lakes. Environ. Pollut..

[18] Nusair S.D., Almasaleekh M.J., Rahman H.R., Alkhatatbeh M. (2019). Environmental exposure of humans to bromide in the Dead Sea area: Measurement of genotoxicy and apoptosis biomarkers. Mutat. Res. Genet. Toxicol. Environ. Mutagen..

[19] Amelia T.S.M., Khalik W.M.A.W.M., Ong M.C., Shao Y., Pan H., Bhubalan K. (2021). Marine microplastics as vectors of major ocean pollutants and its hazards to the marine ecosystem and humans. Prog. Earth Planet. Sci..

[20] Ding T., Wei L., Hou Z., Li J., Zhang C., Lin D. (2022). Microplastics altered contaminant behavior and toxicity in natural waters. J. Hazard. Mater..

[21] Hahladakis J.N., Velis C.A., Weber R., Iacovidou E., Purnell P. (2018). An overview of chemical additives present in plastics: Migration, release, fate and environmental impact during their use, disposal and recycling. J. Hazard. Mater..

[22] Saad D. (2023). Why Microplastics are exceptional contaminants?. Advances and Challenges in Microplastics.

[23] Hu K., Yang Y., Zuo J., Tian W., Wang Y., Duan X., Wang S. (2022). Emerging microplastics in the environment: Properties, distributions, and impacts. Chemosphere.

[24] Vedolin M.C., Teophilo C.Y.S., Turra A., Figueira R.C.L. (2018). Spatial variability in the concentrations of metals in beached microplastics. Mar. Pollut. Bull..

[25] Liu G., Dave P.H., Kwong R.W.M., Wu M., Zhong H. (2021). Influence of microplastics on the mobility, bioavailability, and toxicity of heavy metals: A review. Bull. Environ. Contam. Toxicol..

[26] Munoz M., Ortiz D., Nieto-Sandoval J., de Pedro Z.M., Casas J.A. (2021). Adsorption of micropollutants onto realistic microplastics: Role of microplastic nature, size, age, and NOM fouling. Chemosphere.

[27] Bucci K., Tulio M., Rochman C.M. (2020). What is known and unknown about the effects of plastic pollution: A meta-analysis and systematic review. Ecol. Appl..

[28] Issac M.N., Kandasubramanian B. (2021). Effect of microplastics in water and aquatic systems. Environ. Sci. Pollut. Res..

[29] Kibria G., Haroon A.K.Y., Rose G., Hossain M.M., Nugegoda D. (2021). Pollution Risks, Impacts and Management: Social, Economic, and Environmental Perspectives.

[30] Su L., Deng H., Li B., Chen Q., Pettigrove V., Wu C., Shi H. (2019). The occurrence of microplastic in specific organs in commercially caught fishes from coast and estuary area of east China. J. Hazard. Mater..

[31] McIlwraith H.K., Kim J., Helm P., Bhavsar S.P., Metzger J.S., Rochman C.M. (2021). Evidence of microplastic translocation in wild-caught fish and implications for microplastic accumulation dynamics in food webs. Environ. Sci. Technol..

[32] Lu Y., Zhang Y., Deng Y., Jiang W., Zhao Y., Geng J., Ding L., Ren H. (2016). Uptake and accumulation of polystyrene microplastics in zebrafish (*Danio rerio*) and toxic effects in liver. Environ. Sci. Technol..

[33] Deng Y., Zhang Y., Lemos B., Ren H. (2017). Tissue accumulation of microplastics in mice and biomarker responses suggest widespread health risks of exposure. Sci. Rep..

[34] McCormick M.I., Chivers D.P., Ferrari M., Blandford M.I., Nanninga G., Richardson C., Fakan E.P., Vamvounis G., Gulizia A.M., Allan B.J. (2020). Microplastic exposure interacts with habitat degradation to affect behaviour and survival of juvenile fish in the field. Proc. R. Soc. B Biol. Sci..

[35] Terra B.F., Araújo F.G., Calza C.F., Lopes R., Teixeira T.P. (2008). Heavy metal in tissues of three fish species from different trophic levels in a tropical brazilian river. Water Air Soil Pollut..

[36] Pereira S., Pinto A.L., Cortes R., Fontaínhas-Fernandes A., Coimbra A.M., Monteiro S.M. (2013). Gill histopathological and oxidative stress evaluation in native fish captured in Portuguese northwestern rivers. Ecotoxicol. Environ. Saf..

[37] Velmurugan B., Selvanayagam M., Cengiz E.I., Unlu E. (2009). Histopathological changes in the gill and liver tissues of freshwater fish, *Cirrhinus mrigala* exposed to dichlorvos. Braz. Arch. Biol. Technol..

[38] Thophon S., Kruatrachue M., Upatham E.S., Pokethitiyook P., Sahaphong S., Jaritkhuan S. (2003). Histopathological alterations of white seabass, *Lates calcarifer*, in acute and subchronic cadmium exposure. Environ. Pollut..

[39] Illing B., Rummer J.L. (2017). Physiology can contribute to better understanding, management, and conservation of coral reef fishes. Conserv. Physiol..

[40] Madliger C.L., Love O.P., Hultine K.R., Cooke S.J. (2018). The conservation physiology toolbox: Status and opportunities. Conserv. Physiol..

[41] Carey R., Migliaccio K.W., Li Y., Schaffer B., Kiker G.A., Brown M.T. (2011). Land use disturbance indicators and water quality variability in the Biscayne Bay Watershed, Florida. Ecol. Indic..

[42] Wikelski M., Cooke S.J. (2006). Conservation physiology. Trends Ecol. Evol..

[43] Bolger T., Connolly P.L. (1989). The selection of suitable indices for the measurement and analysis of fish condition. J. Fish. Biol..

[44] Mozsár A., Boros G., Sály P., Antal L., Nagy S.S. (2015). Relationship between Fulton’s condition factor and proximate body composition in three freshwater fish species. J. Appl. Ichthyol..

[45] Mello L.G.S., Rose G.A. (2005). Seasonal cycles in weight and condition in Atlantic cod (*Gadus morhua* L.) in relation to fisheries. ICES J. Mar. Sci..

[46] Ravikumar T., Neethiselvan N., Jayakumar N., Sudhan C., Umamaheswari T., Padmavathy P. (2023). Length-weight relationships and Fulton’s condition factor (K) for 29 demersal reef fishes caught by longline. Thalassas.

[47] Leão-Buchir J., Folle N.M.T., de Souza T.L., Brito M.P., de Oliveira E.C., de Almeida Roque A., Ramsdorf W.A., Fávaro L.F., Garcia J.R.E., Esquivel L. (2021). Effects of trophic 2,2′,4,4′-tetrabromodiphenyl ether (BDE-47) exposure in Oreochromis niloticus: A multiple biomarkers analysis. Environ. Toxicol. Pharmacol..

[48] Lambert Y., Dutil J.D. (1997). Condition and energy reserves of Atlantic cod (*Gadus morhua*) during the collapse of the northern Gulf of St. Lawrence stock. Can. J. Fish. Aquat. Sci..

[49] Bruslé J., Anadon G.G. (1996). The structure and function of fish liver. Fish Morphology.

[50] Dobrzycka-Krahel A., Bogalecka M. (2022). The Baltic Sea under anthropopressure—The sea of paradoxes. Water.

[51] Szymczycha B., Zaborska A., Bełdowski J., Kuliński K., Beszczyńska-Möller A., Kędra M., Pempkowiak J., Sheppard C. (2019). Chapter 4—The Baltic Sea. World Seas: An Environmental Evaluation.

[52] Mutton A.F.A., Couper A.D. (2024). Baltic Sea.

[53] Dargahi B. (2023). Environmental impacts of shallow water mining in the Baltic Sea. Front. Mar. Sci..

[54] Lehmann A., Myrberg K., Post P., Chubarenko I., Dailidiene I., Hinrichsen H.H., Hüssy K., Liblik T., Meier H.E., Lips U. (2022). Salinity dynamics of the Baltic Sea. Earth Syst. Dyn..

[55] Schmidt N., Garate-Olaizola M., Laurila A. (2024). Acclimatizing laboratory-reared hatchling cod (*Gadus morhua*) to salinity conditions in the Baltic Sea. Aquaculture.

[56] Störmer O., Schernewski G., Hofstede J., Neumann T. (2011). Climate change impacts on coastal waters of the Baltic Sea. Global Change and Baltic Coastal Zones.

[57] Lampe R. (1996). Küsten und Küstenschutz in Mecklenburg-Vorpommern. Z. Erdkundeunterricht.

[58] Gogina M., Zettler M.L. (2010). Diversity and distribution of benthic macrofauna in the Baltic Sea: Data inventory and its use for species distribution modelling and prediction. J. Sea Res..

[59] HELCOM (2003). The Baltic marine environment 1999–2002. Baltic Sea Environment Proceedings.

[60] Storie J., Suškevičs M., Nevzati F. (2010). Evidence on the impact of Baltic Sea ecosystems on human health and well-being: A systematic map. Environ. Evid..

[61] HELCOM (2010). Ecosystem health of the Baltic Sea 2003–2007. Baltic Sea Environment Proceedings.

[62] Asmala E., Saikku L., Vienonen S. (2011). Import–export balance of nitrogen and phosphorus in food, fodder and fertilizers in the Baltic Sea drainage area. Sci. Total Environ..

[63] HELCOM (2018). Input of nutrients by the seven biggest rivers in the Baltic Sea region. Baltic Sea Environment Proceedings.

[64] Büngener L., Postila H., Löder M.G.J., Laforsch C., Ronkanen A., Heiderscheidt E. (2023). The fate of microplastics from municipal wastewater in a surface flow treatment wetland. Sci. Total Environ..

[65] Piskuła P., Astel A.M. (2022). Microplastics occurrence in two mountainous rivers in the lowland area—A case study of the Central Pomeranian Region, Poland. Microplastics.

[66] Dereszewska A., Krasowska K., Popek M. (2023). Microplastics in harbor seawaters: A case study in the port of Gdynia. Baltic Sea. Sustainability.

[67] Graca B., Szewc K., Zakrzewska D., Dołęga A., Szczerbowska-Boruchowska M. (2017). Sources and fate of microplastics in marine and beach sediments of the Southern Baltic Sea—A preliminary study. Environ. Sci. Pollut. Res..

[68] Narloch I., Gackowska A., Wejnerowska G. (2022). Microplastic in the Baltic Sea: A review of distribution processes, sources, analysis methods and regulatory policies. Environ. Pollut..

[69] Mishra A., Buhhalko N., Lind K., Lips I., Liblik T., Väli G., Lips U. (2022). Spatiotemporal variability of microplastics in the eastern Baltic Sea. Front. Mar. Sci..

[70] Chubarenko I., Esiukova E., Zobkov M., Isachenko I. (2022). Microplastics distribution in bottom sediments of the Baltic Sea Proper. Mar. Pollut. Bull..

[71] Urban-Malinga B., Zalewski M., Jakubowska A., Wodzinowski T., Malinga M., Pałys B., Dąbrowska A. (2020). Microplastics on sandy beaches of the southern Baltic Sea. Mar. Pollut. Bull..

[72] Piskuła P., Astel A., Pawlik M. (2025). Microplastics in seawater and fish acquired from the corresponding fishing zones of the Baltic Sea. Mar. Pollut. Bull..

[73] Białowąs M., Jonko-Sobuś K., Pawlak J., Polak-Juszczak L., Dąbrowska A., Urban-Malinga B. (2022). Plastic in digestive tracts and gills of cod and herring from the Baltic Sea. Sci. Total Environ..

[74] Sainio E., Lehtiniemi M., Set¨al¨a O. (2021). Microplastic ingestion by small coastal fish in the northern Baltic Sea. Finland. Mar. Pollut. Bull..

[75] Beer S., Garm A., Huwer B., Dierking J., Nielsen T.G. (2018). No increase in marine microplastic concentration over the last three decades—A case study from the Baltic Sea. Sci. Total Environ..

[76] Ojaveer E., Kalejs M. (2010). Ecology and long-term forecasting of sprat (*Sprattus sprattus balticus*) stock in the Baltic Sea: A review. Rev. Fish. Biol. Fish..

[77] Kautsky L., Kautsky N., Mathieson A.C., Nienhuis P.H. (2000). The Baltic Sea, Including Bothnian Sea and Bothnian Bay. World 24: Intertidal and Littoral Ecosystems.

[78] Ojaveer H., Jaanus A., MacKenzie B.R., Martin G., Olenin S., Radziejewska T., Telesh I., Zettler M.T., Zaiko A. (2010). Status of biodiversity in the Baltic Sea. PLoS ONE.

[79] MacKenzie B.R., Gislason H., Möllmann C., Köster F.W. (2007). Impact of 21st century climate change on the Baltic Sea fish community and fisheries. Glob. Change Biol..

[80] Eero M. (2012). Reconstructing the population dynamics of sprat (*Sprattus sprattus balticus*) in the Baltic Sea in the 20th century. ICES J. Mar. Sci..

[81] Aro E. (2002). Fish migration studies in the Baltic Sea: A historical review. ICES Mar. Sci. Symp..

[82] Pawlikowski B., Komar-Szymczak K. (2018). Selected physicochemical and quality indicators of herring from fishery areas in south Baltic. Postęp. Tech. Przetwórstwa Spoż..

[83] Moyano M., Illing B., Polte P., Kotterba P., Zablotski Y., Gröhsler T., Hüdepohl P., Cooke S.J., Peck M.A. (2020). Linking individual physiological indicators to the productivity of fish populations: A case study of Atlantic herring. Ecol. Indic..

[84] Peck M.A., Alheit J., Bertrand A., Catalán I.A., Garrido S., Moyano M., Rykaczewski R.R., Takasuka A., van der Lingen C.D. (2021). Small pelagic fish in the new millennium: A bottom-up view of global research effort. Prog. Oceanogr..

[85] Megrey B.A., Rose K.A., Klumb R.A., Hay D.E., Werner F.E., Eslinger D.E., Smith S.L. (2007). A bioenergetics-based population dynamics model of Pacific herring (*Clupea harengus pallasi*) coupled to a lower trophic level nutrient–phytoplankton–zooplankton model: Description, calibration, and sensitivity analysis. Ecol. Model..

[86] Cooke S.J., Sack L., Franklin C.E., Farrell A.P., Beardall J., Wikelski M., Chown S.L. (2013). What is conservation physiology? Perspectives on an increasingly integrated and essential science. Conserv. Physiol..

[87] Mieszkowska N., Genner M.J., Hawkins S.J., Sims D.W. (2009). Atlantic cod: Impacts of climate change and commercial fishing. Adv. Mar. Biol..

[88] Drinkwater K.F. (2005). The response of Atlantic cod (*Gadus morhua*) to future climate change. ICES J. Mar. Sci..

[89] Magnussen E. (2011). Food and feeding habits of cod (*Gadus morhua*) on the Faroe Bank. ICES J. Mar. Sci..

[90] Ojaveer E., Kalejs M. (2008). On ecosystem-based regions in the Baltic Sea. J. Mar. Syst..

[91] Orio A., Bergström U., Casini M., Erlandsson M., Eschbaum R., Hüssy K., Lehmann A., Ložys L., Ustups D., Florin A.B. (2017). Characterizing and predicting the distribution of Baltic Sea flounder (*Platichthys flesus*) during the spawning season. J. Sea Res..

[92] Nissling A., Dahlman G. (2010). Fecundity of flounder, Pleuronectes flesus, in the Baltic Sea—Reproductive strategies in two sympatric populations. J. Sea Res..

[93] Froese R., Pauly D. (2004). FishBase.

[94] Gibson S.E., Alexander D., Biesecker D., Fisher R.R., Guhathakurta M., Hudson H., Thompson B.J., Habbal S.R., Esser R., Hollweg J.V., Iseberg P.A. (1999). Modeling CMEs in three dimensions using an analytic MHD model. Solar Wind Nine.

[95] Pampoulie C., Skirnisdottir S., Olafsdottir G., Helyar S., Thorsteinsson V., Jónsson S., Fréchet A., Durif C.M.F., Sherman S., Lampart-Kałużniacka M. (2014). Genetic structure of the lumpfish *Cyclopterus lumpus* across the North Atlantic. ICES J. Mar. Sci..

[96] Piskuła P., Astel A.M. (2024). Occurrence of microplastics in commercial fishes from aquatic ecosystems of northern Poland. Ecohydrol. Hydrobiol..

[97] Hidalgo-Ruz V., Gutow L., Thompson R.C., Thiel M. (2012). Microplastics in the marine environment: A review of the methods used for identification and quantification. Environ. Sci. Technol..

[98] Crawford C.B., Quinn B., Crawford C.B., Quinn B. (2017). 10—Microplastic identification techniques. Microplastic Pollutants.

[99] Zobkov M.B., Esiukova E.E. (2018). Microplastics in a marine environment: Review of methods for sampling, processing, and analyzing microplastics in water, bottom sediments, and coastal deposits. Oceanology.

[100] Wang Q., Zhu X., Hou C., Wu Y., Teng J., Zhang C., Tan H., Shan E., Zhang W., Zhao J. (2021). Microplastic uptake in commercial fishes from the Bohai Sea, China. Chemosphere.

[101] European Commission (2013). Monitoring Guidance for Marine Litter in European Seas.

[102] Piskuła P., Astel A.M. (2023). Microplastics in commercial fishes and by-catch from selected FAO Major Fishing Areas of the southern Baltic Sea. Animals.

[103] Blackwell B.G., Brown M.L., Willis D.W. (2000). Relative weight (Wr) status and current use in fisheries assessment and management. Rev. Fish. Sci..

[104] Lusher A.L., McHugh M., Thompson R.C. (2013). Occurrence of microplastics in the gastrointestinal tract of pelagic and demersal fish from the English Channel. Mar. Pollut. Bull..

[105] Ogonowski M., Wenman V., Danielsson S., Gorokhova E. (2017). Ingested Microplastic Is Not Correlated to HOC Concentrations in Baltic Sea Herring.

[106] Lenz R., Enders K., Beer S., Sørensen T.K., Stedmon C.A. (2016). Analysis of microplastic in the stomachs of herring and cod from the North Sea and Baltic Sea. DTU Aqua Natl. Inst. Aquat. Resour..

[107] Kennedy A.B., Sankey W., Riall H. (1898). The Thermal Efficiency of Steam Engines. Minutes Proc. Inst. Civ. Eng..

[108] Wickham H. (2016). ggplot2: Elegant Graphics for Data Analysis.

[109] Davidson D., Marshall C.T. (2010). Are morphometric indices accurate indicators of stored energy in herring *Clupea harengus*?. J. Fish Biol..

[110] Óskarsson G.J., Kjesbu O.S., Slotte A. (2002). Predictions of realised fecundity and spawning time in Norwegian spring-spawning herring (*Clupea harengus*). J. Sea Res..

[111] McPherson L.R., Slotte A., Kvamme C., Meier S., Marshall C.T. (2011). Inconsistencies in measurement of fish condition: A comparison of four indices of fat reserves for Atlantic herring (*Clupea harengus*). ICES J. Mar. Sci..

[112] Pascoe E.S. (2018). Quantifying Interannual Variability in the Condition of Young-of-Year Pacific Herring (*Clupea pallasi*) in the Strait of Georgia, BC. Ph.D. Thesis.

[113] Arula T., Ojaveer H., Shpilev H. (2012). Individual fecundity of the autumn spawning Baltic herring *Clupea harengus membras* L.. Estonian J. Ecol..

[114] Lind Y., Huovila T., Käkelä R. (2018). A retrospective study of fatty acid composition in Baltic herring (*Clupea harengus membras*) caught at three locations in the Baltic Sea (1973–2009). ICES J. Mar. Sci..

[115] Ojaveer H., Tomkiewicz J., Arula T., Klais R. (2015). Female ovarian abnormalities and reproductive failure of autumn-spawning herring (*Clupea harengus membras*) in the Baltic Sea. ICES J. Mar. Sci..

[116] Vainikka A., Mollet F., Casini M., Gårdmark A. (2009). Spatial variation in growth, condition and maturation reaction norms of the Baltic herring *Clupea harengus membras*. Mar. Ecol. Prog. Ser..

[117] Persson M. (2010). Changes in Condition of Herring (Clupea harengus) in Swedish Coastal Waters.

[118] Bucholtz R.H., Tomkiewicz J., Nyengaard J.R., Andersen J.B. (2013). Oogenesis, fecundity and condition of Baltic herring (*Clupea harengus* L.): A stereological study. Fish. Res..

[119] Podolska M., Horbowy J. (2003). Infection of Baltic herring (*Clupea harengus membras*) with *Anisakis simplex* larvae, 1992–1999: A statistical analysis using generalized linear models. ICES J. Mar. Sci..

[120] Strzelczak A., Balejko J., Szymczak M., Witczak A. (2021). Effect of protein denaturation temperature on rheological properties of Baltic Herring (*Clupea harengus membras*) muscle tissue. Foods.

[121] Tserkova F. (2013). Growth parameters of the Black Sea sprat (*Sprattus sprattus* L.) during the period November 2010–March 2012 along the Bulgarian Black Sea coast. Bulg. J. Agric. Sci..

[122] Panayotova M. (2001). Growth Parameters of the Black Sea Sprat (*Sprattus sprattus* L.) During the Period 1998–2000 Along the Bulgarian Black Sea Coast. Proc. Inst. Oceanol..

[123] Vitale F., Mittermayer F., Krischansson B., Johansson M., Casini M. (2015). Growth and maturity of sprat (*Sprattus sprattus*) in the Kattegat and Skagerrak, eastern North Sea. Aquat. Living Resour..

[124] Brendeland Ø. (2022). Spatial Variability in Life History Traits of Sprat (*Sprattus sprattus*) in Norwegian Fjords Suggests Low Mixing of Adults Between the Fjords. Master’s Thesis.

[125] Nadolna-Ałtyn K., Szostakowska B., Podolska M. (2018). Sprat (*Sprattus sprattus*) as a possible source of invasion of marine predators with *Contracaecum osculatum* in the southern Baltic Sea. Russ. J. Mar. Biol..

[126] Keinänen M., Käkelä R., Ritvanen T., Myllylä T., Pönni J., Vuorinen P.J. (2017). Fatty acid composition of sprat (*Sprattus sprattus*) and herring (*Clupea harengus*) in the Baltic Sea as potential prey for salmon (*Salmo salar*). Helgol. Mar. Res..

[127] ICES CM (2001). ACFM:01. Report of the Working Group on the Assessment of Northern Shelf Demersal Stocks.

[128] De Vries A.N., Govoni D., Árnason S.H., Carlsson P. (2020). Microplastic ingestion by fish: Body size, condition factor and gut fullness are not related to the amount of plastics consumed. Mar. Pollut. Bull..

[129] Chouinard G.A., Swain D.P. (2002). Depth-dependent variation in condition and length-at-age of Atlantic cod (*Gadus morhua*) in the southern Gulf of St. Lawrence. Can. J. Fish. Aquat. Sci..

[130] Lilly G.R., Shelton A., Brattey J., Cadigan N., Murphy E.F., Stansbury D.E., Davis M.B., Morgan M.J. (1999). An Assessment of the cod Stock in NAFO Divisions 2J + 3KL. In *NAFO SCR Doc. 99-28*; N4084. https://www.nafo.int/Portals/0/PDFs/sc/1999/scr99-028.pdf.

[131] ICES CM (2002). ACFM:01. Report of the Working Group on the Assessment of Demersal Stocks in the North Sea and Skagerrak.

[132] Kjesbu O.S. (1989). The spawning activity of cod, *Gadus morhua* L.. J. Fish Biol..

[133] Lloret J., Rätz H.J. (2000). Condition of cod (*Gadus morhua*) off Greenland during 1982–1998. Fish. Res..

[134] Rätz H.J., Stein M., Lloret J. (1999). Variation in growth and recruitment of Atlantic cod (*Gadus morhua*) off Greenland during the second half of the twentieth century. J. Northw. Atl. Fish. Sci..

[135] Stansbury D.E. An Assessment of the Cod Stock in NAFO Divisions 3NO. In *NAFO SCR Doc. 01/72*; N4450; 2001; p. 64. https://www.nafo.int/Portals/0/PDFs/sc/2001/scr01-072.pdf.

[136] Mion M., Thorsen A., Vitale F., Dierking J., Herrmann J.P., Huwer B., von Dewitz B., Casini M. (2018). Effect of fish length and nutritional condition on the fecundity of distressed Atlantic cod *Gadus morhua* from the Baltic Sea. J. Fish Biol..

[137] Kraus G., Pelletier D., Dubreuil J., Möllmann C., Hinrichsen H.H., Bastardie F., Vermard Y., Mahévas S. (2009). A model-based evaluation of Marine Protected Areas: The example of eastern Baltic cod (*Gadus morhua callarias* L.). ICES J. Mar. Sci..

[138] Rummel C.D., Löder M.G.J., Fricke N., Lang T., Griebeler E.M., Janke M., Gerdts G. (2016). Plastic ingestion by pelagic and demersal fish from the North Sea and Baltic Sea. Mar. Pollut. Bull..

[139] Kerambrun E., Henry F., Cornille V., Courcot L., Amara R. (2013). A combined measurement of metal bioaccumulation and condition indices in juvenile European flounder, *Platichthys flesus*, from European estuaries. Chemosphere.

[140] Amara R., Selleslagh J., Billon G., Minier C. (2009). Growth and condition of 0-group European flounder, *Platichthys flesus* as indicator of estuarine habitat quality. Hydrobiologia.

[141] Henry F., Filipuci I., Billon G., Courcot L., Kerambrun E., Amara R. (2012). Metal concentrations, growth and condition indices in European juvenile flounder (*Platichthys flesus*) relative to sediment contamination levels in four Eastern English Channel estuaries. J. Environ. Monit..

[142] Mendes C.V.R. (2019). Factors Affecting Early Life Patterns of the European Flounder *Platichthys flesus* in a Nursery Habitat. Ph.D. Thesis.

[143] Freitas V., Santos D., Silva D.M., Cunha J., Rodrigues S.M., Neves V., Rocha E., Martinho F., Ramos S. (2024). Spatial and temporal patterns of gonadal maturation and spawning in European flounder *Platichthys flesus* at its southern continental edge. Fish. Res..

[144] Kleinkauf A., Connor L., Swarbreck D., Levene C., Walker P., Johnson P.J., Leah R.T. (2004). General condition biomarkers in relation to contaminant burden in European flounder (*Platichthys flesus*). Ecotoxicol. Environ. Saf..

[145] Kopecka J., Pempkowiak J. (2008). Temporal and spatial variations of selected biomarker activities in flounder (*Platichthys flesus*) collected in the Baltic proper. Ecotoxicol. Environ. Saf..

[146] Jokinen H., Wennhage H., Lappalainen A., Ådjers K., Rask M., Norkko A. (2015). Decline of flounder (*Platichthys flesus* (L.)) at the margin of the species’ distribution range. J. Sea Res..

[147] Nissling A., Larsson R. (2018). Population specific sperm production in European flounder *Platichthys flesus*: Adaptation to salinity at spawning. J. Fish Biol..

[148] Umaru J.A., Annune P.A., Cheikyula J.O., Okomoda V.T. (2015). Some biometric parameters of four selected fish species in Doma Dam, Nasarawa State, Nigeria. Int. J. Aquac..

[149] Roots O., Schramm K.W., Simmm M., Henkelmann B., Lankov A. (2006). Polychlorinated dibenzo-p-dioxins and dibenzofurans in Baltic herring and sprat in the north-eastern part of the Baltic Sea. Proc. Estonian. Acad. Sci. Biol..

[150] Dos Santos Schmidt T.C., Hay D.E., Sundby S., Devine J.A., Óskarsson G.J., Slotte A., Wuenschel M.J., Lajus D., Johannessen A., van Damme C.J.G. (2021). Adult body growth and reproductive investment vary markedly within and across Atlantic and Pacific herring: A meta-analysis and review of 26 stocks. Rev. Fish Biol. Fish..

[151] Wheeler J.P., Purchase C.F., Macdonald P.D.M., Fill R., Jacks L., Wang H., Ye C. (2009). Temporal changes in maturation, mean length-at-age, and condition of spring-spawning Atlantic herring (*Clupea harengus*) in Newfoundland waters. ICES J. Mar. Sci..

[152] Frantzen S., Måge A., Iversen S.A., Julshamn K. (2011). Seasonal variation in the levels of organohalogen compounds in herring (*Clupea harengus*) from the Norwegian Sea. Chemosphere.

[153] Cardinale M., Casini M., Arrhenius F. (2002). The influence of biotic and abiotic factors on the growth of sprat (*Sprattus sprattus*) in the Baltic Sea. Aquat. Living Resour..

[154] Simm M., Roots O., Kotta J., Lankov A., Henkelmann B., Shen H., Schramm K.W. (2006). PCDD/Fs in sprat (*Sprattus sprattus balticus*) from the Gulf of Finland, the Baltic Sea. Chemosphere.

[155] Moore C., Lynch D., Clarke M., Officer R., Mills J., Louis-Defour J., Brophy D. (2019). Age verification of north Atlantic sprat. Fish. Res..

[156] Casarsa L., Diez M.J., Madirolas A., Cabreira A.G., Buratti C.C. (2019). Morphometric description of schools from two different stocks of the southernmost sprat *Sprattus fuegensis*. Fish. Res..

[157] Van Beveren E., Bonhommeau S., Fromentin J.M., Bigot J.L., Bourdeix J.H., Brosset P., Roos D., Saraux C. (2014). Rapid changes in growth, condition, size and age of small pelagic fish in the Mediterranean. Mar. Biol..

[158] Sinovcic G., Franicevic M., Zorica B., Ciles-Kec V. (2004). Length-weight and length-length relationships for 10 pelagic fish species from the Adriatic Sea (Croatia). J. Appl. Ichthyol..

[159] Kalaycı F., Samsun N., Bilgin S., Samsun O. (2007). Length-weight relationship of 10 fish species caught by bottom trawl and midwater trawl from the Middle Black Sea, Turkey. Turk. J. Fish. Aquat. Sci..

[160] Kasapoğlu N. (2018). Age, growth, and mortality of exploited stocks: Anchovy, Sprat, Mediterranean Horse Mackerel, Whiting, and Red Mullet in the Southeastern Black Sea. Aquat. Sci. Eng..

[161] Stancheva M., Merdzhanova A., Petrova E., Petrova D. (2013). Heavy metals and proximate composition of Black Sea sprat (*Sprattus sprattus*) and goby (*Neogobius melanostomus*). Bulg. J. Agric. Sci..

[162] Zuyev G.V., Bondarev V.A., Samotoi I.V. (2018). Local overfishing of the Black Sea sprat (*Sprattus sprattus*: Clupeidae, Pisces) and intraspecies differentiation. Mar. Biol. J..

[163] Solberg I., Røstad A., Kaartvedt S. (2015). Ecology of overwintering sprat *(Sprattus sprattus*). Prog. Oceanogr..

[164] Svedäng H., Hornborg S. (2017). Historic changes in length distributions of three Baltic cod (*Gadus morhua*) stocks: Evidence of growth retardation. Ecol. Evol..

[165] Cardinale M., Modin J. (1999). Changes in size-at-maturity of Baltic cod (*Gadus morhua*) during a period of large variations in stock size and environmental conditions. Fish. Res..

[166] Kraus G., Müller A., Trella K., Köuster F.W. (2000). Fecundity of Baltic cod: Temporal and spatial variation. J. Fish Biol..

[167] Pachur M.E., Horbowy J. (2013). Food composition and prey selection of cod, *Gadus morhua* (Actinopterygii: Gadiformes: Gadidae), in the southern Baltic Sea. Acta Ichthyol. Piscat..

[168] Kraus G., Tomkiewicz J., Diekmann R., Köster F.W. (2008). Seasonal prevalence and intensity of follicular atresia in Baltic cod *Gadus morhua callarias L.*. J. Fish Biol..

[169] Svedäng H., Hornborg S. (2014). Selective fishing induces density-dependent growth. Nat. Commun..

[170] Florin A.B., Höglund J. (2008). Population structure of flounder (*Platichthys flesus*) in the Baltic Sea: Differences among demersal and pelagic spawners. Heredity.

[171] Napierska D., Baršienė J., Mulkiewicz E., Podolska M., Rybakovas A. (2009). Biomarker responses in flounder *Platichthys flesus* from the Polish coastal area of the Baltic Sea and applications in biomonitoring. Ecotoxicology.

[172] Polak-Juszczak L. (2013). Trace metals in flounder, *Platichthys flesus* (Linnaeus, 1758), and sediments from the Baltic Sea and the Portuguese Atlantic coast. Environ. Sci. Pollut. Res..

[173] Borg J.P.G., Westerbom M., Lehtonen H. (2014). Sex-specific distribution and diet of *Platichthys flesus* at the end of spawning in the northern Baltic Sea. J. Fish Biol..

[174] Sahin T., Gunes E., Aydin I., Polat H. (2008). Reproductive characteristics and egg development in flounder (*Pleuronectes flesus luscus*) in the Southern Black Sea. IJA.

[175] Lampart-Kaluzniacka M., Heese T. (2000). Morphological characteristics of South Baltic lumpfish, *Cyclopterus lumpus* l., 1758. Acta Ichthyol. Piscat..

[176] Engebretsen S., Aldrin M., Lunde L., Austad M., Rafoss T., Danielsen O.R., Lindhom A., Boissonnot L., Jansen P.A. (2024). Condition factor tailored to lumpfish (*Cyclopterus lumpus*) used as cleaner fish in salmonid farms. Aquac. Rep..

[177] Engebretsen S., Aldrin M., Qviller L., Stige L.C., Rafoss T., Danielsen O.R., Lindhom A., Jansen P.A. (2023). Salmon lice (*Lepeophtheirus salmonis*) in the stomach contents of lumpfish (*Cyclopterus lumpus*) sampled from Norwegian fish farms: Relationship between lice grazing and operational conditions. Aquaculture.

[178] Whittaker B.A., Consuegra S., Garcia de Leaniz C. (2015). Genetic and phenotypic differentiation of lumpfish (*Cyclopterus lumpus*) across the North Atlantic: Implications for conservation and aquaculture. PeerJ.

[179] Lovell J.M., Findlay M.M., Moate R.M., Pilgrim D.A. (2005). The polarization of inner ear ciliary bundles from a scorpaeniform fish. J. Fish Biol..

[180] Cardinale M., Arrhenius F. (2000). Decreasing weight-at-age of Atlantic herring (*Clupea harengus*) from the Baltic Sea between 1986 and 1996: A statistical analysis. ICES J. Mar. Sci..

[181] Casini M., Rouyer T., Bartolino V., Larson N., Grygiel W. (2014). Density-dependence in space and time: Opposite synchronous variations in population distribution and body condition in the Baltic Sea sprat (*Sprattus sprattus*) over three decades. PLoS ONE.

[182] Shelton P.A., Sinclair A.F., Chouinard G.A., Mohn R., Duplisea D.E. (2006). Fishing under low productivity conditions is further delaying recovery of Northwest Atlantic cod (*Gadus morhua*). Can. J. Fish. Aquat. Sci..

[183] Casini M., Cardinale M., Arrhenius F. (2004). Feeding preferences of herring (*Clupea harengus*) and sprat (*Sprattus sprattus*) in the southern Baltic Sea. ICES J. Mar. Sci..

[184] Heikinheimo O. (2011). Interactions between cod, herring and sprat in the changing environment of the Baltic Sea: A dynamic model analysis. Ecol. Modell..

[185] Casini M., Cardinale M., Hjelm J. (2006). Inter-annual variation in herring, *Clupea harengus*, and sprat, *Sprattus sprattus*, condition in the central Baltic Sea: What gives the tune?. Oikos.

[186] Aps R., Lassen H. (2010). Recovery of depleted Baltic Sea fish stocks: A review. ICES J. Mar. Sci..

[187] Olsson J., Bergström L., Gårdmark A. (2012). Abiotic drivers of coastal fish community change during four decades in the Baltic Sea. ICES J. Mar. Sci..

[188] Yao C.L., Somero G.N. (2012). The impact of acute temperature stress on hemocytes of invasive and native mussels (*Mytilus galloprovincialis* and *Mytilus californianus*): DNA damage, membrane integrity, apoptosis and signaling pathways. J. Exp. Biol..

[189] Willig M.R., Kaufam D.M., Stevens R.D. (2003). Latitudinal gradients of biodiversity: Pattern, process, scale, and synthesis. Annu. Rev. Ecol. System..

[190] Fonds M., Cronie R., Vethaak A.D., Van Der Puyl P. (1992). Metabolism, food consumption and growth of plaice (*Pleuronectes platessa*) and flounder (*Platichthys flesus*) in relation to fish size and temperature. Neth. J. Sea Res..

[191] Neill W.H., Miller J.M., Van Der Veer H.W., Winemiller K.O. (1994). Ecophysiology of marine fish recruitment: A conceptual framework for understanding interannual variability. Neth. J. Sea Res..

[192] Fincham J.I., Rijnsdorp A.D., Engelhard G.H. (2013). Shifts in the timing of spawning in sole linked to warming sea temperatures. J. Sea Res..

[193] Neuheimer A., Thresher R., Lyle J., Semmens J.M. (2011). Tolerance limit for fish growth exceeded by warming waters. Nature Clim. Change.

[194] Castellani C., Edwards M. (2017). Marine Plankton: A Practical Guide to Ecology, Methodology, and Taxonomy.

[195] Steffensen J.F. (2005). Respiratory systems and metabolic rates. Fish Physiol..

[196] Aydin İ., Firidin Ş., Öztürk R., Alemdağ M., Terzi̇ Y., Eroğlu O. (2024). Genetically Distinct European Flounder (*Platichthys flesus* L.) Matriline in the Black Sea. Thalassas.

[197] Soliño L., Vidal-Liñán L., Pérez P., García-Barcelona S., Baldó F., Gago J. (2022). Microplastic occurrence in deep-sea fish species Alepocephalus bairdii and Coryphaenoides rupestris from the Porcupine Bank (North Atlantic). Sci. Total Environ..

[198] Maaghloud H., Houssa R., Ouansafi S., Bellali F., El Bouqdaoui K., Charouki N., Fahde A. (2020). Ingestion of microplastics by pelagic fish from the Moroccan Central Atlantic coast. Environ. Pollut..

[199] Jonathan M.P., Sujitha S.B., Rodriguez-Gonzalez F., Villegas L.E., Hernández-Camacho C.J., Sarkar S.K. (2021). Evidences of microplastics in diverse fish species off the Western Coast of Pacific Ocean, Mexico. Ocean Coast. Manag..

[200] Digka N., Tsangaris C., Torre M., Anastasopoulou A., Zeri C. (2018). Microplastics in mussels and fish from the Northern Ionian Sea. Mar. Pollut. Bull..

[201] Güven O., Gökdağ K., Jovanović B., Kıdeyş A.E. (2017). Microplastic litter composition of the Turkish territorial waters of the Mediterranean Sea, and its occurrence in the gastrointestinal tract of fish. Environ. Pollut..

[202] Abadi Z.T.R., Abtahi B., Grossart H.P., Khodabandeh S. (2021). Microplastic content of Kutum fish, Rutilus frisii kutum in the southern Caspian Sea. Sci. Total Environ..

[203] Ding J., Ju P., Ran Q., Li J., Jiang F., Cao W., Zhang J., Sun C. (2023). Elder fish means more microplastics? Alaska pollock microplastic story in the Bering Sea. Sci. Adv..

[204] Gao S., Yan K., Liang B., Shu R., Wang N., Zhang S. (2023). The different ways microplastics from the water column and sediment accumulate in fish in Haizhou Bay. Sci. Total Environ..

[205] Abbasi A., Sadeghi P., Abadi Z.T.R. (2023). Characterization of microplastics in digestive tract of commercial fish species from the Oman Sea. Mar. Pollut. Bull..

[206] Schernewski G., Radtke H., Hauk R., Baresel C., Olshammar M., Osinski R., Oberbeckmann S. (2020). Transport and Behavior of Microplastics Emissions from Urban Sources in the Baltic Sea. Front. Environ. Sci..

[207] Cardinale M., Casini M., Arrhenius F., Håkansson N. (2003). Diel spatial distribution and feeding activity of herring (*Clupea harengus*) and sprat (*Sprattus sprattus*) in the Baltic Sea. Aquat. Living Resour..

[208] Kulatska N., Woods P.J., Elvarsson B., Bartolino V. (2021). Size-selective competition between cod and pelagic fisheries for prey. ICES J. Mar. Sci..

[209] Bošković N., Joksimović D., Perošević-Bajčeta A., Peković M., Bajt O. (2022). Distribution and characterization of microplastics in marine sediments from the Montenegrin coast. J. Soils Sediments.

[210] Rodríguez-Rey M., Whittaker B. (2023). The global ecological niche of lumpfish (*Cyclopterus lumpus*) and predicted range shifts under climate change. Hydrobiologia.

[211] McGoran A.R., Clark P.F., Morritt D. (2017). Presence of microplastic in the digestive tracts of European flounder, *Platichthys flesus*, and European smelt, Osmerus eperlanus, from the River Thames. Environ. Pollut..

[212] Jabeen K., Su L., Li J., Yang D., Tong C., Mu J., Shi H. (2017). Microplastics and mesoplastics in fish from coastal and fresh waters of China. Environ. Pollut..

[213] Koongolla J.B., Lin L., Pan Y.F., Yang C.P., Sun D.R., Liu S., Xu X.R., Maharana D., Huang J.S., Li H.X. (2020). Occurrence of microplastics in gastrointestinal tracts and gills of fish from Beibu Gulf, South China Sea. Environ. Pollut..

[214] Keerthika K., Padmavathy P., Rani V., Jeyashakila R., Aanand S., Kutty R. (2022). Contamination of microplastics, surface morphology and risk assessment in beaches along the Thoothukudi coast, Gulf of Mannar region. Environ. Sci. Pollut. Res..

[215] Feng Z., Zhang T., Li Y., He X., Wang R., Xu J., Gao G. (2019). The accumulation of microplastics in fish from an important fish farm and mariculture area, Haizhou Bay, China. Sci. Total Environ..

[216] Pan Z., Zhang C., Wang S., Sun D., Zhou A., Xie S., Xu G., Zou J. (2021). Occurrence of microplastics in the gastrointestinal tract and gills of fish from Guangdong, South China. J. Mar. Sci. Eng..

[217] Jaafar N., Azfaralariff A., Musa S.M., Mohamed M., Yusoff A.H., Lazim A.M. (2021). Occurrence, distribution and characteristics of microplastics in gastrointestinal tract and gills of commercial marine fish from Malaysia. Sci. Total Environ..

[218] Zheng S., Tang S., Wang W.X. (2024). Microplastics and nanoplastics induced differential respiratory damages in tilapia fish Oreochromis niloticus. J. Hazard. Mater..

[219] Trani A., Mezzapesa G., Piscitelli L., Mondelli D., Nardelli L., Belmonte G., Toso A., Piraino S., Panti C., Baini M. (2023). Microplastics in water surface and in the gastrointestinal tract of target marine organisms in Salento coastal seas (Italy, Southern Puglia). Environ. Pollut..

[220] Seetapan K., Prommi T.O. (2023). Microplastics in commercial fish digestive tracts from freshwater habitats in Northern Thailand. Ecol. Montenegrina.

[221] Atamanalp M., Köktürk M., Uçar A., Duyar H.A., Özdemir S., Parlak V., Esenbuğa N., Alak G. (2021). Microplastics in tissues (brain, gill, muscle and gastrointestinal) of *Mullus barbatus* and *Alosa immaculata*. Arch. Environ. Contam. Toxicol..

[222] Barboza L.G.A., Lopes C., Oliveira P., Bessa F., Otero V., Henriques B., Raimundo J., Caetano M., Vale C., Guilhermino L. (2020). Microplastics in wild fish from North East Atlantic Ocean and its potential for causing neurotoxic effects, lipid oxidative damage, and human health risks associated with ingestion exposure. Sci. Total Environ..

[223] Su Y., Lin H.C. (2023). Analyses of microplastics in the digestive tract of bottom-trawled fishes in Southwest Taiwan. Reg. Stud. Mar. Sci..

[224] De Sales-Ribeiro C., Brito-Casillas Y., Fernandez A., Caballero M. (2020). An end to the controversy over the microscopic detection and effects of pristine microplastics in fish organs. Sci. Rep..

[225] Ding J., Zhang S., Razanajatovo R.M., Zou H., Zhu W. (2018). Accumulation, tissue distribution, and biochemical effects of polystyrene microplastics in the freshwater fish red tilapia (*Oreochromis niloticus*). Environ. Pollut..

[226] Jovanović B., Gökdağ K., Güven O., Emre Y., Whitley E.M., Kideys A.E. (2018). Virgin microplastics are not causing imminent harm to fish after dietary exposure. Mar. Pollut. Bull..

[227] Fuglem B., Jirillo E., Bjerkås I., Kiyono H., Nochi T., Yuki Y., Raida M., Fischer U., Koppang E.O. (2010). Antigen-sampling cells in the salmonid intestinal epithelium. Dev. Comp. Immunol..

[228] Løvmo S.D., Speth M.T., Repnik U., Koppang E.O., Griffiths G.W., Hildahl J.P. (2017). Translocation of nanoparticles and Mycobacterium marinum across the intestinal epithelium in zebrafish and the role of the mucosal immune system. Dev. Comp. Immunol..

[229] Handy R.D., Henry T.B., Scown T.M., Johnston B.D., Tyler C.R. (2008). Manufactured nanoparticles: Their uptake and effects on fish—A mechanistic analysis. Ecotoxicology.

[230] Guerrera M.C., Aragona M., Porcino C., Fazio F., Laurà R., Levanti M., Montalbano G., Germanà G., Abbate F., Germanà A. (2021). Micro and nano plastics distribution in fish as model organisms: Histopathology, blood response and bioaccumulation in different organs. Appl. Sci..

[231] Tepe Y., Aydın H., Ustaoğlu F., Kodat M. (2024). Occurrence of microplastics in the gastrointestinal tracts of four most consumed fish species in Giresun, the Southeastern Black Sea. Environ. Sci. Pollut. Res. Int..

[232] Suaria G., Avio C.G., Mineo A., Lattin G.L., Magaldi M.G., Belmonte G., Moore C.J., Regoli F., Aliani S. (2016). The Mediterranean Plastic Soup: Synthetic polymers in Mediterranean surface waters. Sci. Rep..

[233] Mondal P., Hoque M.S., Rahman M.A., Hasan M.M., Chakma S., Islam M.S., Shahjahan M. (2024). Occurrence, characteristics and distribution of microplastics in commercial marine fishes of the Bay of Bengal. Mar. Pollut. Bull..

[234] Cordova M.R., Riani E., Shiomoto A. (2020). Microplastics ingestion by blue panchax fish (*Aplocheilus* sp.) from Ciliwung Estuary, Jakarta, Indonesia. Mar. Pollut. Bull..

[235] Yin J., Ju Y., Qian H., Wang J., Miao X., Zhu Y., Zhou L., Ye L. (2022). Nanoplastics and microplastics may be damaging our livers. Toxics.

[236] Sun X., Li Q., Zhu M., Liang J., Zheng S., Zhao Y. (2017). Ingestion of microplastics by natural zooplankton groups in the northern South China Sea. Mar. Pollut. Bull..

[237] Botterell Z.L.R., Beaumont N., Dorrington T., Steinke M., Thompson R.C., Lindeque P.K. (2019). Bioavailability and effects of microplastics on marine zooplankton: A review. Environ. Pollut..

[238] Clere I.K., Ahmmed F., Remoto P., Fraser-Miller S.J., Gordon K.C., Komyakova V., Allan B.J.M. (2022). Quantification and characterization of microplastics in commercial fish from southern New Zealand. Mar. Pollut. Bull..

[239] Kasamesiri P., Thaimuangphol W. (2020). Microplastics ingestion by freshwater fish in the Chi river, Thailand. Int. J. Geomate.

[240] Hamed M., Martyniuk C.J., Lee J.-S., Shi H., Sayed A.L.-D. (2023). Distribution, abundance, and composition of microplastics in market fishes from the Red and Mediterranean seas in Egypt. J. Sea Res..

[241] Santonicola S., Volgare M., Di Pace E., Mercogliano R., Cocca M., Raimo G., Colavita G. (2023). Research and characterization of fibrous microplastics and natural microfibers in pelagic and benthic fish species of commercial interest. Ital. J. Food Saf..

[242] Scacco U., Mancini E., Marcucci F., Tiralongo F. (2022). Microplastics in the deep: Comparing dietary and plastic ingestion data between two Mediterranean bathyal opportunistic feeder species, *Galeus melastomus* and *Coelorinchus caelorhincus*, through stomach content analysis. J. Mar. Sci. Eng..

[243] Carrillo-Barragán P., Fitzsimmons C., Lloyd-Hartley H., Tinlin-Mackenzie A., Scott C., Sugden H. (2024). Fifty-year study of microplastics ingested by brachyuran and fish larvae in the central English North Sea. Environ. Pollut..

[244] Plastics Europe (2019). Plastics—The Facts 2019. An Analysis of European Plastic Production, Demand and Waste Data. *Plastics Europe*. https://plasticseurope.org/wp-content/uploads/2021/10/2019-Plastics-the-facts.pdf.

[245] McKeen L.W. (2017). Environmentally friendly polymers. Plast. Des. Libr..

[246] Cózar A., Echevarría F., González-Gordillo J.I., Irigoien X., Úbeda B., Hernández-León S., Palma Á.T., Navarro S., García-de-Lomas J., Ruiz A. (2014). Plastic debris in the open ocean. Proc. Natl. Acad. Sci. USA.

[247] Galloway T.S. (2015). Micro- and nano-plastics and human health. Mar. Anthropog. Litter.

[248] Herbinger C.M., Friars G.W. (1991). Correlation between condition factor and total lipid content in Atlantic salmon, *Salmo salar* L.. Aquac. Res..

[249] Chellappa S., Huntingford F.A., Strang R.H.C., Thomson R.Y. (1995). Condition factor and hepatosomatic index as estimates of energy status in male three-spined stickleback. J. Fish Biol..

[250] Barrett C.J., Johnson M.L., Hall N.J., Hull S.L. (2016). The first use of Fulton’s K for assessing and comparing the conditions of inter-tidal fish populations. Mar. Ecol..

[251] Imsland A.K., Reynolds P., Hangstad T.A., Jónsdóttir Ó., Noble T., Wilson M., Mackie J.A., Elvegård T.A., Urskog T.C., Mikalsen B. (2018). Feeding behaviour and growth of lumpfish (*Cyclopterus lumpus* L.) fed with feed blocks. Aquac. Res..

[252] Imsland A.D.K., Frogg N., Stefansson S.O., Reynolds P. (2019). Improving sea lice grazing of lumpfish (*Cyclopterus lumpus* L.) by feeding live feeds prior to transfer to Atlantic salmon (*Salmo salar* L.) net-pens. Aquaculture.

[253] Imsland A.K.D., Berg M.S., Haugland G.T., Eliasen K. (2022). Comparing body density of lumpfish (*Cyclopterus lumpus*) to different operational welfare indicators. Fishes.

[254] Imsland A.K.D., Reynolds P., Lorentzen M., Eilertsen R.A., Micallef G., Tvenning R. (2020). Improving survival and health of lumpfish (*Cyclopterus lumpus* L.) by the use of feed blocks and operational welfare indicators (OWIs) in commercial Atlantic salmon cages. Aquaculture.

[255] Rajasilta M., Laine P., Paranko J. (2011). Current growth, fat reserves and somatic condition of juvenile Baltic herring (*Clupea harengus membras*) reared in different salinities. Helgol. Mar. Res..

[256] Neuenfeldt S., Bartolino V., Orio A., Andersen K.H., Andersen N.G., Niiranen S., Bergström U., Ustups D., Kulatska N., Casini M. (2020). Feeding and grow of Atlantic cods (*Gadus morhua* L.) in the east Baltic Sea under environmental change. ICES J. Mar. Sci..

[257] Eriksson H., Albert J., Albert S., Warren R., Pakoa K., Andrew N. (2017). The role of fish and fisheries in recovering from natural hazards: Lessons learned from Vanuatu. Environ. Sci. Policy.

[258] Bryhn A.C., Bergek S., Bergström U., Casini M., Dahlgren E., Ek C., Hjelm J., Königson S., Ljungberg P., Lundström K. (2022). Which factors can affect the productivity and dynamics of cod stocks in the Baltic Sea, Kattegat and Skagerrak?. Ocean Coast. Manag..

[259] Ighwela K.A., Ahmad A.B., Abol-Munafi A.B. (2014). The selection of viscerosomatic and hepatosomatic indices for the measurement and analysis of *Oreochromis niloticus* condition fed with varying dietary maltose levels. Int. J. Fauna Biol..

[260] Sadekarpawar S., Parikh P. (2013). Gonadosomatic and hepatosomatic indices of freshwater fish *Oreochromis mossambicus* in response to a plant nutrient. World J. Zool..

[261] Dambo A., Solomon S.G., Ayuba V.O., Okayi R.G. (2021). Study on condition factor and hepatosomatic index of *Bagrus bayad* (Forsskal, 1775) and *Synodontis nigrita* (*Valenciennes*, 1840) from Kangimi Reservoir, Kaduna State, Nigeria. BAJOPAS.

[262] Popović N.T., Čižmek L., Babić S., Strunjak-Perović I., Čož-Rakovac R. (2023). Fish liver damage related to the wastewater treatment plant effluents. Environ. Sci. Pollut. Res..

[263] Austin B. (1998). The effects of pollution on fish health. J. Appl. Microbiol..

[264] Vethaak A.D., Jol J.G. (1996). Diseases of flounder *Platichthys flesus* in Dutch coastal and estuarine waters, with particular reference to environmental stress factors. I. Epizootiology of gross lesions. DAO.

[265] Lang T., Wosniok W., Baršienė J., Broeg K., Kopecka J., Parkkonen J. (2006). Liver histopathology in Baltic flounder (*Platichthys flesus*) as indicator of biological effects of contaminants. Mar. Pollut. Bull..

[266] Schubert S., Keddig N., Gerwinski W., Neukirchen J., Kammann U., Haarich M., Hanel R., Theobald N. (2016). Persistent organic pollutants in Baltic herring (*Clupea harengus*)—An aspect of gender. Environ. Monit. Assess..

[267] Sweidan A.H., El-Bendary N., Hegazy O.M., Hassanien A.E., Snasel V. (2015). Water pollution detection system based on fish gills as a biomarker. Procedia Comput. Sci..

[268] Pandey S., Parvez S., Ansari R.A., Ali M., Kaur M., Hayat F., Ahmad F., Raisuddin S. (2008). Effects of exposure to multiple trace metals on biochemical, histological and ultrastructural features of gills of a freshwater fish, *Channa punctata* Bloch. Chem. Biol. Interact..

[269] Aslam S., Yousafzai A.M. (2017). Chromium toxicity in fish: A review article. J. Entomol. Zool. Stud..

[270] Rohani M.F. (2023). Pesticides toxicity in fish: Histopathological and hemato-biochemical aspects—A review. Emerg. Contam..

[271] Da Cruz A.L., Prado T.M., da Silva Maciel L., Couto R.D. (2015). Environmental effects on the gills and blood of *Oreochromis niloticus* exposed to rivers of Bahia, Brazil. Ecotoxicol. Environ. Saf..

[272] Steinberg C.E.W. (2018). Diets and digestive tracts—‘your food determines your intestine’. Aquatic Animal Nutrition.

[273] Kleinow K.M., James M.O., Schlenk D., Benson W.H. (2001). Response of the teleost gastrointestinal system to xenobiotics. Target Organ Toxicity in Marine and Freshwater Teleosts.

[274] Bandowe B.A.M., Bigalke M., Boamah L., Nyarko E., Saalia F.K., Wilcke W. (2014). Polycyclic aromatic compounds (PAHs and oxygenated PAHs) and trace metals in fish species from Ghana (West Africa): Bioaccumulation and health risk assessment. Environ. Int..

[275] Ajima M.N.O., Nnodi P.C., Ogo O.A., Adaka G.S., Osuigwe D.I., Njoku D.C. (2015). Bioaccumulation of heavy metals in Mbaa River and the impact on aquatic ecosystem. Environ. Monit. Assess..

[276] Filgueiras A.V., Preciado I., Cartón A., Gago J. (2020). Microplastic ingestion by pelagic and benthic fish and diet composition: A case study in the NW Iberian shelf. Mar. Pollut. Bull..

[277] Lopes C., Ambrosino A.C., Figueiredo C., Caetano M., Santos M.M., Garrido S., Raimundo J. (2023). Microplastic distribution in different tissues of small pelagic fish of the Northeast Atlantic Ocean. Sci. Total Environ..

[278] Menéndez D., Blanco-Fernandez C., Machado-Schiaffino G., Ardura A., Garcia-Vazquez E. (2023). High microplastics concentration in liver is negatively associated with condition factor in the Benguela hake *Merluccius polli*. Ecotoxicol. Environ. Saf..

[279] Sbrana A., Valente T., Scacco U., Bianchi J., Silvestri C., Palazzo L., de Lucia G.A., Valerani C., Ardizzone G., Matiddi M. (2020). Spatial variability and influence of biological parameters on microplastic ingestion by *Boops boops* (L.) along the Italian coasts (Western Mediterranean Sea). Environ. Pollut..

[280] Welden N.A.C., Cowie P.R. (2016). Long-term microplastic retention causes reduced body condition in the langoustine, *Nephrops norvegicus*. Environ. Pollut..

[281] Critchell K., Hoogenboom M.O. (2018). Effects of microplastic exposure on the body condition and behaviour of planktivorous reef fish (*Acanthochromis polyacanthus*). PLoS ONE.

[282] Wright S.L., Rowe D., Thompson R.C., Galloway T.S. (2013). Microplastic ingestion decreases energy reserves in marine worms. Curr. Biol..

[283] Watts A.J.R., Urbina M.A., Corr S., Lewis C., Galloway T.S. (2015). Ingestion of plastic microfibers by the crab *Carcinus maenas* and its effect on food consumption and energy balance. Environ. Sci. Technol..

[284] Lohmann R. (2017). Microplastics are not important for the cycling and bioaccumulation of organic pollutants in the oceans—But should microplastics be considered POPs themselves?. Integr. Environ. Assess. Manag..

[285] Compa M., Ventero A., Iglesias M., Deudero S. (2018). Ingestion of microplastics and natural fibres in *Sardina pilchardus* (Walbaum, 1792) and *Engraulis encrasicolus* (Linnaeus, 1758) along the Spanish Mediterranean coast. Mar. Pollut. Bull..

