# Supplementary material for: Well-Being of the Baltic Herring and Bycatch Fish Species from FAO Major Fishing Areas 27 According to Microplastic Pollution
Source: Animals (Basel). 2025 Aug 13;15(16):2381. doi: 10.3390/ani15162381 (PMC12382631; doi:10.3390/ani15162381)
Supplement: Supplementary file 1 [file animals-15-02381-s001.zip › animals-3782362-supplementary.pdf]

Table S1. Spearman's correlation coefficient values between total length and total mass of the fish according to species.

Species	Spearman's ranked correlation coefficient total length [cm] vs total mass [g]
baltic herring	(n = 127) r_S = 0.63, $r_{S\_crit\ (p=0.05)}$ < 0.165
flounder	(n = 46) r_S = 0.70, $r_{S\_crit\ (p=0.05)}$ = 0.246
baltic cod	(n = 29) r_S = 0.78, $r_{S\_crit\ (p=0.05)}$ = 0.312
lumpfish	(n = 17) r_S = 0.82, $r_{S\_crit\ (p=0.05)}$ = 0.414
long-spined bullhead	(n = 6) r_S = 0.88, $r_{S\_crit\ (p=0.05)}$ = 0.829
sprat	(n = 27) r_S = 0.66, $r_{S\_crit\ (p=0.05)}$ = 0.324

Note: r_{S_crit} – critical value of the Spearman's ranked correlation coefficient
